# A review of engraftment assessments following fecal microbiota transplant

**DOI:** 10.1080/19490976.2025.2525478

**Published:** 2025-07-02

**Authors:** Chloe Herman, Bridget M. Barker, Thais F. Bartelli, Vidhi Chandra, Rosa Krajmalnik-Brown, Mary Jewell, Le Li, Chen Liao, Florencia McAllister, Khemlal Nirmalkar, Joao B. Xavier, J. Gregory Caporaso

**Affiliations:** a Pathogen and Microbiome Institute, Northern Arizona University, Flagstaff, AZ, USA; b School of Informatics, Computing and Cyber Systems, Northern Arizona University, Flagstaff, AZ, USA; c Department of Genetics, University of Texas MD Anderson Cancer Center, Houston, TX, USA; d Biodesign Center for Health Through Microbiomes, Arizona State University, Tempe, AZ, USA; e School of Sustainable Engineering and the Built Environment, Arizona State University, Tempe, AZ, USA; f Independent Author, Salt Lake City, UT, USA; g Program for Computational and Systems Biology, Memorial Sloan-Kettering Cancer Center, New York, NY, USA; h Department of Gastrointestinal Medical Oncology, University of Texas MD Anderson Cancer Center, Houston, TX, USA; i Department of Immunology, The University of Texas MD Anderson Cancer Center, Houston, TX, USA; j Department of Biological Sciences, Northern Arizona University, Flagstaff, AZ, USA

**Keywords:** Fecal microbiota transplant, stool transplant, microbiome, bacteriotherapy, intestinal microbiota transplant, engraftment, bioinformatics

## Abstract

Fecal Microbiota Transplant (FMT) is a treatment for recurrent *Clostridium difficile* infections and is being explored for other clinical applications, from alleviating digestive and neurological disorders, to restoring microbiomes impacted by cancer treatment. Quantifying the extent of engraftment following an FMT is important in understanding a recipient’s response to treatment. Engraftment and clinical response need to be investigated independently to evaluate an FMT’s role (or lack thereof) in achieving a clinical response. Standardized bioinformatics methodologies for quantifying engraftment extent would not only improve assessment and understanding of FMT outcomes, but also facilitate comparison of FMT results and protocols across studies. Here we review FMT studies, integrating three concepts from microbial ecology as framework to discuss how these studies approached assessing engraftment extent: 1) *Community Coalescence* investigates microbiome shifts following FMT engraftment, 2) *Indicator Features* tracks specific microbiome features as a signal of engraftment, and 3) *Resilience* examines how resistant post-FMT recipients’ microbiomes are to reverting back to baseline. These concepts explore subtly different questions about the microbiome following FMT. Taken together, they provide holistic insight into how an FMT alters a recipient’s microbiome composition and provide a clear framework for quantifying and communicating about microbiome engraftment.

## Introduction

The gut microbiome impacts diverse aspects of our health, including host immune response;^1,2^ inflammatory bowel diseases such as Crohn’s Disease and ulcerative colitis^3,4^; metabolic diseases such as obesity and diabetes;^3,5^ autoimmune diseases;^6–8^ diverse cancers;^9–12^ and even neurological conditions through the gut-brain axis.^13–17^

The many links between the gut microbiome and human health have raised the promise of altering the microbiome to improve health.^18,19^ Fecal Microbiota Transplant (FMT) is a medical procedure that transfers microbes from fecal material, or fecal material as a whole, from a donor to a recipient, for example, through endoscopies, retention enemas,^20,21^ or oral capsules^22^ in attempt to restore or modulate a recipient’s gut microbiome. The donor could be a “healthy” individual, an individual who previously responded to treatment similar to one the recipient is undergoing or any individual (including the same individual at an earlier time) whose microbiome researchers want to transplant. Before donation, the donated microbiome is typically screened for specific bacterial pathogens, multidrug resistant organisms (MDROs), viruses, parasites, and general health metrics, including, but not limited to, history of gastrointestinal illness, history of autoimmune disorders, or BMI >30 kg/m^2.22^

FMT has shown strong efficacy for the treatment of recurrent *Clostridium difficile* infection (rCDI) when other treatments, such as antibiotics, have failed to prevent the infection from reccuring.^23^ Given these findings, in 2013 the US Food and Drug Administration (FDA) issued guidance stating that FMTs may be used as a treatment for rCDI if standard treatments had failed. At the time, there were significant obstacles to implementing FMTs, including how the safety of the donated microbiome should be assessed, how the material should be prepared for transplantation, deciding upon the most suitable administration route, and designing other aspects of the treatment protocol.^22^ In 2022, the first FMT microbiota product, a single dose enema, was approved by the FDA for treating recurrent *C. difficile* infection,^24^ and more recently, in 2023, the first oral FMT microbiota product was approved.^25^ In contrast to the 2013 guidance, the 2022 approval uses stool from pre-approved donated microbiomes, making FMTs for rCDI more accessible and standardized.^24^

Although there are currently no FDA approved FMT products for applications outside of treating recurrent *C. difficile* infections, preliminary successes have been reported for FMT in improving Immune Checkpoint Inhibitors (ICPI) responses,^26–28^ remediation of tumor growth,^29^ treating irritable bowel syndrome,^27,30^ improving aging hallmarks,^31^ diabetes,^32^ behavioral and digestive symptoms associated with autism spectrum disorder,^33,34^ Alzheimer’s disease,^35^ reestablishing gut microbiota after allogeneic hematopoietic cell transplant,^36–39^ and more. While FMTs have shown success in treating a wide variety of problems related to the gut microbiome, there remain many challenges with assessing the success of individual FMTs and understanding the specific microbial changes they catalyze.

When microbiome engraftment has been explicitly considered in the literature, a wide range of analyses and perspectives have been applied for its quantification, making it difficult for researchers, especially those new to the FMT field, to know where to begin in terms of assessing engraftment in their work. Additionally, this inconsistency in methodology makes it challenging to compare findings across studies. The central aim of this review is therefore to facilitate a better understanding of how engraftment has been assessed in the FMT literature to date and to contextualize this work in a conceptual framework. We will begin by defining important terminology and concepts that are useful for discussing microbiome engraftment in a consistent manner.

## Microbiome data types and features

The units of observation in microbiome studies, and the terminology used by researchers, vary with the technology used to profile the microbiome. In small subunit ribosomal RNA amplicon (SSU rRNA, or 16S rRNA) studies, the currently preferred unit of study is the amplicon sequence variant,^40^ or ASV – a unique sequence determined after data quality control. Before this approach was widespread, SSU rRNA studies grouped sequence variants into operational taxonomic units (OTUs), generally by clustering sequences at some percent identity. In a microbiome metagenomics survey, sequence reads, assembled contigs, or metagenome-assembled genomes (MAGs), are generally assigned taxonomy, and a taxonomic unit such as genus, species, or strain is used as the unit of observation. Alternatively, in a microbiome metagenomics or metatranscriptomics survey, functional characteristics of a microbiome, such as observed or active genes or pathways, could be the units of observation. In a mass-spectrometry-based metaproteomics or metabolomics study, individual peptides, proteins, or small molecules could be the units of observation.

Most studies reviewed here used 16S amplicon data to assess engraftment, but many of the same methods could be applied to metagenomics data (e.g., to assess the functional potential of the transplanted microbiome and/or enable higher taxonomic resolution variants,^41–43^ metatranscriptomics and/or metaproteomics data (to assess whether transplanted microbiomes perform the same functions in the recipients as they do in the hosts), or even metabolomics data (e.g., to assess change in postbiotics). Increased taxonomic resolution through microbiome metagenomics enables tracking specific donated microbiome features with higher specificity, for example, at the species or strain level. Additionally, if an FMT benefits the recipient based on the beneficial functional traits that are transferred, not the specific microbes,^43^ then metagenomics data would support the assessment of functional trait transfer. However, metagenomics can be problematic in low microbial biomass samples due to human DNA contamination, and the higher sequencing costs sometimes compromise study design by requiring infrequent temporal sampling.

Despite the difference across these data types and units of observation, many of the downstream analyses are very similar. In the following sections, we use the intentionally general term *feature* to refer to any of these units of observation. The term *feature table* will be used to describe a table of feature frequencies (counts), relative abundances, or presence/absence information on a per sample basis, for all samples in a given study.

## Clinical response and its relationship with microbiome engraftment

FMT engraftment and clinical response are often discussed together, but these concepts should be investigated independently. In this section, we will define **clinical response** and **engraftment extent** and discuss their relationship as explored in the literature.

**Figure 1.**
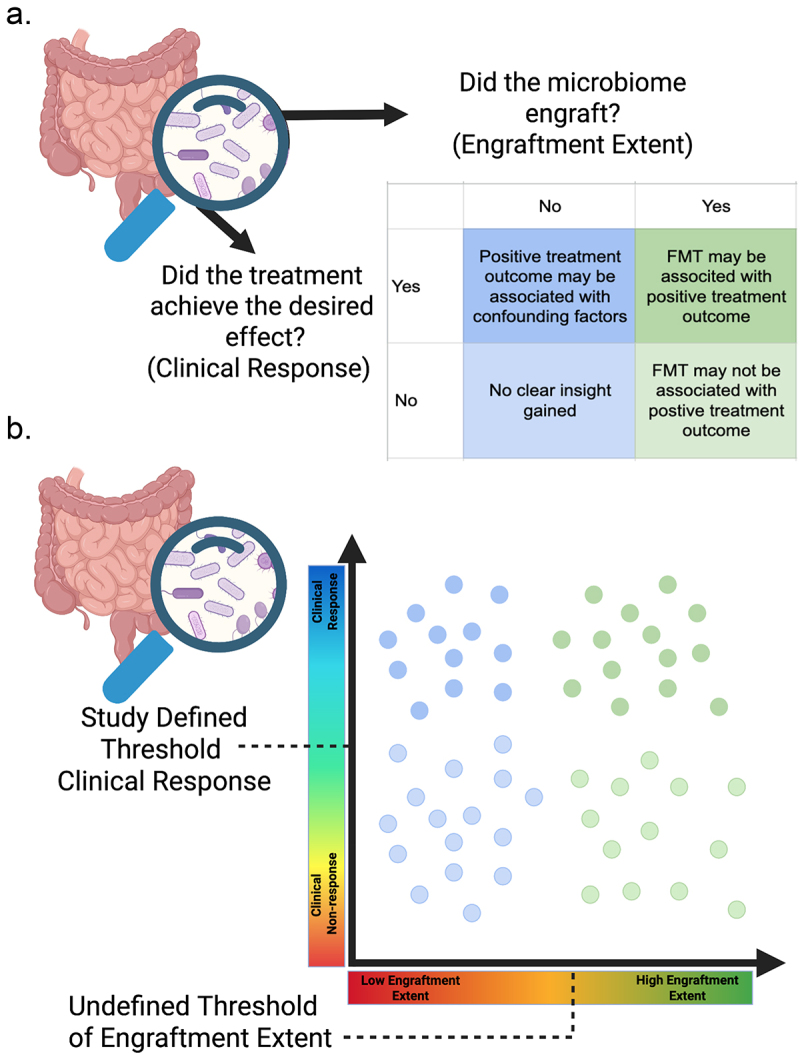
Assessing the success of FMT. a) When investigating outcomes of an FMT, two questions should be asked: did the microbiome engraft, and did the treatment achieve the desired outcome? b) In reality, these questions are not binary. *Clinical response* is commonly a spectrum of response and *engraftment extent* is similarly a spectrum of how much the donated microbiome influences the recipient microbiome. Created in BioRender. Caporaso, G. (2025).

If a treatment achieves the desired outcome (as defined by study-specific criteria), we define that as **clinical response**, while **clinical non-response** is defined here as the treatment not achieving the desired outcome. It is important to note that in clinical trials, clinical responses are usually pre-defined by clinical endpoints outlined in the initial study design. The definition of clinical response thus varies with the disease of interest, but for many diseases, consensus exists among clinicians on what constitutes clinical success. For example, Khanna et al.^44^ considered no occurrences of a *C. difficile* infection for 6 months after the FMT intervention a successful clinical outcome, and van Lingen et al.^45^ used the Mayo score for assessing severity of ulcerative colitis.^45^ FMT studies often track a patient’s response to the FMT by tracking disease status, although this does not address whether the FMT treatment actually resulted in the transplant and colonization of a microbiome. A **clinical non-responder** (someone who received the FMT but had no improvement in disease status or symptoms) could have not responded because FMT is not an effective treatment for their condition, or because the FMT itself was unsuccessful, in which case we are not able to assess whether FMT is an effective treatment for their condition. Clinical response is generally not a simplistic binary variable (response or nonresponse), but rather a continuous measure with thresholds for success established per-study (Figure 1(b)).

Microbiome engraftment following FMT, similar to clinical response, is also not a binary variable, but rather a continuous measure of how much the donor microbiome influences the recipient’s microbiome. We refer to this spectrum of engraftment as **engraftment extent** (Figure 1(b)).

Some clinical studies consider FMT a success when the desired clinical outcome is achieved, but microbiome engraftment should be considered independently of the clinical success of the treatment. This distinction will enable researchers to detangle the effect of the FMT engraftment from other factors. Similarly, in some model organism studies, researchers use FMTs in an attempt to mimic the human microbiome of specific diseases in mice. However, it is common for these studies to have limited engraftment assessment,^46,47^ which makes it difficult to evaluate whether observed phenotypes are due to modeled gut microbiome or other factors.

Clinical response and its relationship with engraftment extent (and other microbiome-level processes) is still an active area of research, and like the definition of clinical response, is likely to vary by disease. Many studies find positive correlation between engraftment signal and clinical response,^33,48^ and in a meta-analysis of 24 FMT cohorts, Ianiro et al.^49^ found a significant relationship between clinical response and increased percentage of donor strain engrafted,^49^ although they did note that the percentage associated with clinical success varied across the cohorts. Other studies have observed a lack of correlation between percent of donor strain engrafted and clinical response,^50–53^ which suggests that relevant microbiome-related factors exist that are not captured in the approach used for quantifying engraftment, or that a high extent of donor strain engraftment is not necessary to achieve clinical success for some diseases. The latter seems to be the case for rCDI,^50,54–56^ where FMT is most developed as a treatment, and the field is now moving toward treatment options composed of specific, known microbes that are most associated with clinical response, and moving away from whole microbiome transfer from fecal material.^49,57^ For other applications of FMT, such as restoring an individual’s gut microbiome following chemotherapy with autoFMT,^11^ microbiome richness following FMT seems to be most associated with clinical success.^11^

A consistent methodology for assessing diverse aspects of engraftment extent will facilitate comparing results across studies and allow us to best understand and communicate what aspects, if any, of the FMT are most impactful and should be optimized to maximize clinical response for a given disease.

## An ecological framework for assessing engraftment extent

Here, we review over 50 primary studies where FMT was used either as a treatment or as a step for replicating microbiomes in model organisms, and which explicitly investigated engraftment. We present the approaches that were applied for assessing engraftment in the context of three ecological concepts, community coalescence, indicator features, and resilience, which we define here.

**Figure 2.**
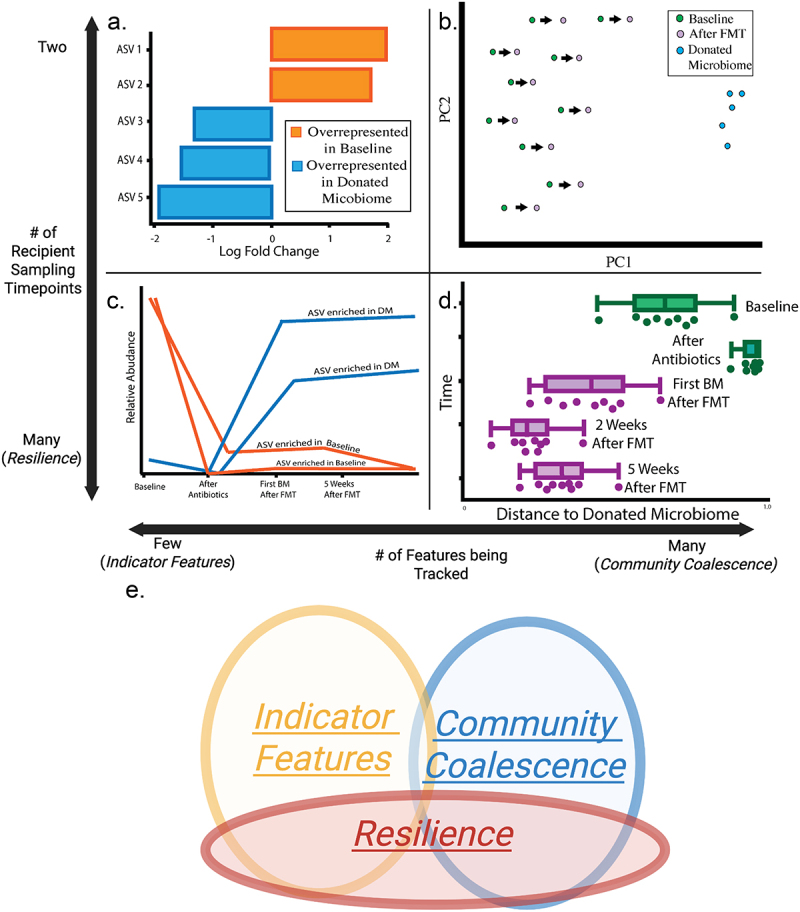
Framework for quantifying and communicating engraftment extent. a) *Indicator feature* tracking between pre- and post FMT samples or between donated microbiome and pre-/post FMT samples highlights which important microbes were transferred from the donated microbiome to the recipient. This approach to assessing engraftment is focused on individual features and their successful or unsuccessful engraftment into the recipient. b) *Community Coalescence* approaches between pre- and post FMT samples or between donated microbiome and recipient pre-/post FMT samples illustrate how the recipient community merges with the donated microbiome. This gives a global view of how the donated microbiome influences the recipient microbiome following FMT. c) Incorporating *resilience* investigation into the *indicator features* concept enables tracking of important features over time. This could be informative for understanding which donated microbiome indicator features engraft long term and possibly reveal indicator features that positively correlate with long term clinical response. In the clinical setting, this combined approach could also provide guidance for re-administration of the FMT if a critical indicator feature was lost after follow-up. d) Combining *community coalescence* and *resilience* concepts facilitates investigation of how the donated microbiome alters the recipient microbiome long term. Allowing for investigation of long-term alterations could provide insight on correlations between donor/recipient similarity and clinical response for various human health conditions, loss of similarity to the donor over time, or long term reestablishment of an individualized microbiome. e) These concepts are overlapping ideas for assessing engraftment, and similar tools may be applied to the data to address these concepts. When applied together, this framework facilitates communication and quantification of whole-microbiome alterations (*Community coalescence*), and specific microbe engraftment (*Indicator features*) following FMT, as well as the long-term influences of both of these concepts (*Resilience*).

*Community Coalescence* provides a global view of microbiome engraftment, by measuring how two microbial communities come together to form a new community (Figure 2(b)).^58^ In the case of an FMT, the two communities that are merging are the recipient’s baseline microbiome and the donated microbiome. *Community Coalescence* should result in a microbiome that is chimeric, meaning that the merger results in a single community of interacting microbes, as opposed to remaining two independent but cohabitating communities.^58^ Furthermore, to illustrate a successful incorporation of the donated microbiome, *Community Coalescence* should be asymmetric, meaning that the recipient’s microbiome after FMT should be more similar to the donated microbiome than to the recipient’s baseline.^58^ To illustrate *Community Coalescence*, the microbiome of the recipient after FMT should shift significantly away from their baseline (pre-treatment) microbiome and toward the donated microbiome in terms of richness, composition, and/or structure of the microbiome. However, the donated microbiome and the recipient’s microbiome will almost certainly not be identical after FMT.

*Indicator Feature* tracking provides a related but more specific view of microbiome engraftment, by monitoring what specific features transferred from a donor to a recipient as a result of FMT (Figure 2(a)). Following a successful FMT, features present in the donated microbiome but absent from the recipient pre-transplant should be observed in the recipient post-transplant. For some FMT applications, notably rCDI, clinical success is defined by a similar measure: a feature present at baseline in the recipient (e.g., *C. difficile*) should be absent in the recipient post-transplant.^44^ Tracking donated microbiome-associated features is one indicator of how effectively the donated microbiome transferred into the recipient following FMT,^42,59^ as opposed to simply introducing a disruption to the recipient’s microbiome (which could be confounded with other perturbations such as antibiotic use).

Precise tracking of indicator features can be difficult with the limited taxonomic resolution of current technologies. Techniques such as strain-profiling with metagenomics data provide higher resolution than, for example, 16S data, but the higher resolution comes at higher cost. Additionally, even if genomically identical organisms were observed in the donated microbiome and in the recipient post-transplant, it is effectively impossible to know whether that organism was present in the recipient prior to transplant without sequencing the full genome of every single microbial cell in the recipient’s gut prior to transplant. For this reason, we strongly recommend against only using indicator features to assess engraftment.

Finally, tracking *Resilience* provides long-term insights into community coalescence (Figure 2(d)) and the presence or absence of indicator features (Figure 2(c)) following FMT intervention. Some methods for microbiome alteration, such as probiotics, often have transient effects on the microbiome,^60–62^ but if the goal of an FMT is microbiome alteration that persists after treatment ends, short and long-term assessment of the alteration is relevant. A resilient FMT would result in a microbiome that does not revert back to the recipient’s baseline microbiome, though long-term similarity to the donated microbiome is likely not essential for positive clinical outcomes (and probably not even the goal as the human gut microbiome is dynamic).^34^ Through our literature review, we have not identified a consensus for how long that resilience should last, and this is almost certainly dependent on the disease being treated; FMT studies differ widely in how long and at how many timepoints the microbiome is assessed post-FMT (Supplemental Data B).

While these concepts overlap with one another, they offer complimentary insight regarding engraftment. Together, they provide a useful framework for communicating about and quantifying different aspects of engraftment by investigating how similar the post-transplant microbiome is to the donated microbiome as a whole, whether specific microbes of interest transferred, and if microbiome alterations persist over time (Figure 2(e)). Depending on the goal of an individual FMT, some of these concepts may be more relevant than others. For example, *Indicator Features* may be of the most interest if the goal of the FMT is to transfer specific microbes from the donor (for example, recent studies have found that engraftment of specific genera are correlated with positive clinical outcomes in rCDI);^50,54–56^ however, *Community Coalescence* might be more relevant if the goal of the FMT is to transfer the whole microbial community (for example, if attempting to model a disease-associated microbiome in a rodent).

Here, we leverage these concepts to relate approaches for assessing microbiome engraftment across studies. Most of the studies we reviewed use one or more approaches to assess engraftment, so our citing a study for a specific approach does not imply that it was the only approach used. Supplemental Table S1 lists the engraftment assessment approaches used by study. Additional discussion on visualization techniques for presenting engraftment results are provided in Supplemental Text 1. Our review will conclude by offering specific recommendations for utilizing this ecological framework in future assessments of microbiome engraftment.

## Engraftment quantification in FMT literature

### Community coalescence

*Community Coalescence* provides a global view of engraftment extent by investigating how the donated microbiome altered the recipient microbiome. In the literature, this was commonly analyzed using three approaches: alpha diversity, beta diversity and source tracking.

#### Alpha diversity

Alpha diversity metrics are able to track a shift in microbiome richness and/or evenness, but similar metric values do not indicate that the same features are present. Thus, they can relate microbiome changes to treatment, but they do not provide specific information about the extent to which a microbiome has engrafted. For example, two microbiome samples could have the same *number* of features present, but none of the same features. Alpha diversity is frequently assessed in FMT studies and is sometimes treated as a measure of *Community Coalescence*, but it does not provide this information. Therefore, we do not discuss alpha diversity in this review, but do include additional discussion in Supplemental Text 1.

#### Beta diversity

Beta diversity is typically used in microbiome research to quantify the dissimilarity of the taxonomic or phylogenetic microbial composition of pairs of samples. This is often measured as a distance: the larger the distance between a pair of samples, the more dissimilar the samples are to each other. There are a wide variety of distance metrics, including Bray Curtis,^63^ Jensen – Shannon divergence,^64^ Unweighted Unifrac distance,^65^ Weighted Unifrac distance,^65^ Jaccard,^66^ Euclidean distances, Donor Similarity Index,^67^ Sorensen’s Similarity Index,^63^ and normalized Kimura 2-parameter.^68^ Beta diversity methods commonly compare the distance from the FMT recipient’s post-FMT gut microbiome to their baseline sample or to the donated microbiome as an assessment of changes induced in the gut microbiome as a result of FMT.

##### Distance of recipient to donated microbiome

The analysis that we most frequently observed for using beta diversity to assess *Community Coalescence* was assessing whether a recipient’s distance to the donated microbiome decreased following FMT by comparing their baseline and post-FMT distances to the donated microbiome. Some studies look at the post-FMT distance to donated microbiome in isolation,^48,69–73^ while most track this over multiple timepoints.^12,33,34,36,41,45,53,56,59,74–79^ Most studies present a significant decrease in distance to the donated microbiome sample following FMT, implying increased similarity.^12,33,34,36,41,59,70,74^ For example, Kang et al.^34^ found that Unweighted Unifrac distance to the donated microbiome decreased after administration of the FMT and concluded high FMT engraftment. In another study, Bloom et al.^75^ did not observe a microbial shift toward the donated microbiome’s composition with FMT, which might be indicative of engraftment failure, but other methods would help to confirm.

##### Distance of recipient to baseline

Another common distance calculated is the recipient’s distance to their baseline, which is also often tracked over multiple timepoints.^29,39,53,70,72,80–83^ This comparison helps inform whether the recipient’s microbiome after FMT intervention has shifted away from their baseline, which is another important element of assessing FMT engraftment extent because this shows that the recipient’s microbiome is shifting in response to the FMT, possibly illustrating high resilience if observed over the study. Gopalakrishnan et al.^80^ used Weighted and Unweighted Unifrac metrics to track distance to baseline over time and noted that recipients who received less frequent doses of FMT (for example, a single dose compared to five doses) remained more similar to their baseline samples at the end of the study relative to recipients who received larger doses, suggesting a dose-dependent response.

##### Distance of recipient to control

Similarly, some studies compared FMT recipients’ distance to a control, such as a Sham FMT group.^54,59,71,74,84–86^ This approach was applied to identify how different a placebo group was to an FMT group, which informs whether changes in the microbiome after the transplant are attributable to FMT engraftment, as opposed to other components of the treatment protocol, such as antibiotic use. Wang et al.^41^ used Bray-Curtis dissimilarity index to compare treatment groups to a control that had received antibiotics but no FMT.

#### Source tracking

Tracking how many features and/or the proportion of features that are transferred from the donated microbiome to the recipient can highlight the successful engraftment of the features of interest.^29,36,37,42,53,54^ One approach for tracking the number of transferred features is to quantify the proportion of features from varying sources, which is often referred to as source tracking. Often FMT researchers are interested in whether specific features come from a donated microbiome, a recipient, or both.^29,36,37,42,53,54^ Some FMT studies also track features that come from neither the donated microbiome nor the recipient,^36,42^ but were possibly recruited by the recipients’ diet or other environmental sources after FMT intervention. (Those features could also be sourced from the donated microbiome or recipient but were present at a level that was not detectable in the relevant source samples.)

SourceTracker^87^ (and more recently MAGEnTa^88^) are software tools for source tracking features in microbiome data, where some samples are defined as sources and other samples are defined as sinks. SourceTracker then reports the relative contributions of the different source sample types to each sink. When used in this way, SourceTracker provides an approach for assessing *Community Coalescence*. SourceTracker also reports on individual features that inform source predictions, and therefore can overlap with the approaches for *Indicator Feature*s.

Aggarwala et al.^89^ defined a method for measuring the percentage of donated microbiome features in the recipient’s microbiome, Proportional Engraftment of Donor Strains (PEDS). They define this as the total number of donated microbiome strains found in the recipient after FMT intervention over the total number of donated microbiome strains.

$$\mathrm{PEDS}=\frac{\mathrm{Total}\;\mathrm{Number}\;\mathrm{of}\;\mathrm{Donated}\;\mathrm{Microbiome}\;\mathrm{Strains}\;\mathrm{in}\;\mathrm{Recipient}}{\mathrm{Total}\;\mathrm{Number}\;\mathrm{of}\;\mathrm{Donated}\;\mathrm{Microbiome}\;\mathrm{Strains}}$$

They also define Proportional Persistence of Recipient Strains (PPRS) as the number of recipient strains that persist in the recipients’ microbiome after FMT intervention.

$$\mathrm{PPRS}=\frac{\mathrm{Total}\;\mathrm{Number}\;\mathrm{of}\;\mathrm{Recipient}\;\mathrm{Strains}\;\mathrm{in}\;\mathrm{Recipient}\;\mathrm{after}\;\mathrm{FMT}}{\mathrm{Total}\;\mathrm{Number}\;\mathrm{of}\;\mathrm{Recipient}\;\mathrm{Strains}}$$

In Aggarwala et al.^89^ the researchers track the gut microbiome of individuals with recurrent *C. difficile* infections before and after FMT intervention. The mean PEDS in the recipients post-FMT was 75%, and the mean PPRS ranged from 15% to 50% for the duration of the 5 year experiment. They reported that 17% PEDS was the threshold for clinical response (ie., no recurrent *C. difficile* infections after FMT intervention).^89^ Similarly, Routy et al.^76^ investigated PEDS but defined it as strain engraftment: the number of engrafted strains after FMT divided by the number of strains in the donated microbiome.

Many studies use methodologies similar to PEDS, PPRS, and SourceTracker (which are currently available tools for source tracking), but use custom code for the individual study. Of the researchers that defined custom methodologies, the most common is to label features based on their source and then investigate the source proportions in FMT recipients. The most common labels were *donor* and *recipient*, but some studies also contain *neither* and/or *both*.^29,36,37,39,42,56^ Gopalakrishnan et al.^80^ slightly modified this by labeling Amplicon Sequence Variant (ASV) sources as mouse, human, or both. They then tracked the percentages of each source in recipient samples throughout their time series. Instead of tracking the proportion over time, Singh et al.^30^ investigated engraftment rate, which they defined as the presence of donated microbiome OTUs found in post-FMT recipient microbiomes that were not observed in the recipient microbiomes at baseline.^30^

Most studies reported an increased percentage of donated microbiome-sourced features in FMT recipients post-FMT relative to baseline. However, Wilson et al.^42^ found that after FMT intervention, there was an increase in features originating from neither the recipient nor the donated microbiome.^42^ This indicated that there may be a microbial community developing following the transplant that is not specifically driven by the donated microbiome or recipient’s microbiome, but they also note that this could represent a failure to detect low abundance taxa in the donated microbiome or recipient’s baseline microbiome.

## Indicator features

Assessing *Community Coalescence* gives a high-level perspective on how the microbiome has changed with FMT. Tracking of specific donated microbiome features is also a popular approach to assessing engraftment that focuses on individual features rather than a summary of the microbiome as a whole. In this concept, which we refer to as *Indicator Features*, specific features are considered to serve as indicators of engraftment. We describe multiple related approaches that differ in how indicator features are identified. Differential Abundance (DA) testing, which encompasses a large variety of methods that identify features whose average abundances are significantly different across groups, is central to this approach, but the specific tests that are applied differ.

Linear models are commonly used for testing differential abundance of *Indicator Features*.^31,42,59,75,77,82,90,91^ Some teams used linear mixed models,^92^ generalized linear models (MaAsLin2),^93,94^ generalized least square models,^95^ and mixed-effects models.^96^ Another team used SourceTracker to identify important species that were indicative of engraftment.^50^ The time points that investigators compared vary; many teams compared the baseline to time points after the FMT intervention,^31,42,59,70,72,75,79,83,84,90^ to the donated microbiome,^71,82^ to a control group,^71,73,85,86,97^ or to an alternative FMT method.^98^ After DA testing is applied, the identified features are commonly tracked over time as an indicator of engraftment.

El-Salhy et al.^99^ used the GA-map Dysbiosis Test^Ⓡ^ ,^100,101^ which is based on a predetermined list of features, and then tracked change in this dysbiosis index before and after FMT. Routy et al.,^76^ used Aldex2^102^ to compare baseline samples to post-FMT-intervention samples.^76^ Some researchers used two-tailed t-tests.^69,81^ Damman et al.^81^ used paired two-tailed t-tests to compare differences in feature abundance between recipients before FMT intervention and the donated microbiome to see if specific features were missing in the recipient prior to FMT treatment. DA testing on microbiome data is still challenging due to the characteristics of microbiome data, including compositionality and sparsity. Traditional distribution tests, such as t-tests, applied to all features in the microbiome feature table are not a reliable way to identify differentially abundant features because large number of comparisons (one for every observed feature) increases the chance of false positives, and this simplistic statistic test can not account for the compositional nature of shifts in the microbiome. Users of these approaches should assess the state of the field when they are ready to run these analyses to be sure they are using up-to-date methods.

Another approach similar to differential abundance testing utilizes important features identified by a predictive linear model. This allows for a model to describe changes in composition or sources over time and for researchers to use the important features from this prediction to track engraftment.^79,103^ Staley et al.,^24^ also found important features by using corr.axes to find OTUs affecting axis position.^54^

### Tracking previously identified indicator features

A closely related approach is to track microbiome features that were determined to be of relevance to the FMT independently of the current study dataset. The distinction between this approach and those previously described is that the features of interest would be derived from relevant studies on other cohorts (or another source, such as model organism studies). While DA testing could still be applied here, more traditional pairwise comparisons focused on the specific features of interest (rather than all the features observed in a data set) are generally more powerful, as they do not require controlling the false discovery rate over very large number of comparisons. We refer to this as tracking “previously identified” indicator features, and the types of features used with this approach can vary widely.^54,92^

DeFilipp et al.^36^ investigated *Clostridiales* as an indicator feature after FMT because previous allo-HCT therapy literature showed that *Clostridiales* is a commensal anaerobe that contributes to intestinal homeostasis, and that a decrease of *Clostridiales* had been positively associated with transplant-related mortality.^38,39^ They performed a Mann-Whitney-U^104^ test on the relative abundance of *Clostridiales* to test for a significant difference across different timepoints.

Similarly, Kang et al.^34^ tracked changes in relative abundance of three genera: *Bifidobacterium, Prevotella* and *Desulfovibrio. Bifidobacterium* and *Prevotella* are commensal bacteria that were found in previous studies to be depleted in children with autism relative to neurotypical controls.^105,106^ *Desulfovibrio* has been reported as both detrimental and commensal in the gut microbiome by different research groups but has been identified as an important bacterial genus in autism studies.^107^ They used a Wilcoxon signed rank test^108^ to track changes over time and found that *Desulfovibrio* changes with FMT were significant and *Bifidobacterium* and *Prevotella* were nearly significant.^33^ In their two-year follow-up study, they compared the log_10_ of relative abundance of *Bifidobacterium, Prevotella* and *Desulfovibrio* from recipient’s baseline sample to time points throughout the experiment using Wilcoxon signed rank test, and they compared recipients to neurotypical controls throughout and after the intervention using Mann-Whitney-U tests.^34^

In another study that tracked previously identified features of interest, the researchers tracked the *Prevotella* to *Bacteroides* ratio, because *Prevotella* and *Bacteroides* were posited to define dominant “enterotypes” that are helpful for clinical diagnoses.^109^ (The enterotype hypothesis is disputed due to significant variation and instability within defined enterotypes, and no distinct boundaries between enterotypes).^110–112^ The team found that the recipients did have an increased *Prevotella* to *Bacteroides* ratio after FMT intervention.^42^

Similarly, van Lier et al.^37^ tracked predicted butyrate-producing bacteria as well as *Blautia* and *Clostridiales* across FMT study timepoints. They found that clinical responders to the FMT seemed to have an increase in *Clostridiales* and other predicted butyrate-producing bacteria relative to the clinical non-responders.^37^

### Tracking loss of indicator features

Another application of using indicator features to assess engraftment is tracking loss of baseline-recipient features, though in some cases we think that it is debatable whether this is an indicator of clinical response or of microbiome engraftment. Important baseline-recipient features can be identified as such with linear or machine learning models, based on differential abundance between donor to recipient or based on previous literature. Tracking the loss of baseline-recipient features is common in rCDI studies, as an important indicator of success is the reduction of *Clostridium* clusters.^50,54,79^ Loss or reduction of *Clostridium* clusters may be an indicator of positive engraftment, but as with the other criteria presented here, engraftment cannot be illustrated by loss of an indicator feature alone. Persistent absence of baseline-recipient features may be a sign of resilience of the recipient’s post-transplant microbiome, even if their microbiome shifts away from the donors (i.e., becomes “personalized”) long term.

## Resilience

Stability of engraftment is an important aspect of FMT intervention. Clinicians often have a checkup with the patient 3–7 days after FMT intervention, and another checkup at 4–8 weeks is recommended.^22^ It can be useful to use these opportunities to assess engraftment extent by testing whether the microbial communities have sustained over time. In probiotic studies, there are often transient features (features that are not observed long-term in the microbiome), which might benefit the recipient temporarily due to the microbe’s metabolic activity, but offer limited long-term changes to the microbiome.^15,60–62^ The goal of an FMT is generally the transition away from an unhealthy microbiome state toward a new-to-the-recipient healthier microbiome state. The changes introduced by the FMT should therefore have some degree of stability. Most FMT studies are longitudinal and track the microbiome over time to investigate the effect of the FMT after the initial intervention. However, there is no consensus in the scientific literature on how long the microbiome needs to be maintained after FMT intervention to qualify as engraftment. The median study length was 65 days (among papers that directly stated the study length, *n* = 51) between the first and last time point sample. The minimum length of these studies was 7 days^9^ and the maximum length was 1,825 days^89^ (Supplemental Data B). Minimal justification for study length was given. Standardization of the FMT follow-up timeline would help to compare FMT engraftment extent across studies. Without standardized follow-ups, it is difficult to determine if an FMT that was tracked for 100 days is as successful as a FMT tracked for 10 days because the 100-day study recipients may lose microbiome similarity to their donor over that time frame, but that same divergence may not be visible after only 10 days.

Despite the lack of standardization for follow-up periods to study *Resilience*, many studies have found that long-term changes following FMTs can shed light on how FMTs impact the human gut. Some studies have found no long term effects of an FMT on the recipient’s microbiome after 18 weeks,^103^ while Wang et al. suggest resilience in the microbiome should be monitored and additional FMTs should be provided to patients to encourage more engraftment.^41^

Other studies found initial similarity to the donor that later diverged into a “personalized microbiome”.^34,78^ For example, Kang et al.^34^ saw that after two years, the post-FMT microbiome of the recipients had relatively large distances to both the donated microbiome and the participants’ baseline microbiome (as assessed by unweighted UniFrac and other beta diversity metrics), and rather looked like a new microbiome state relative to those previously observed in the study. The authors considered the engraftment successful because the recipients retained an increased community richness at the two year follow-up. Weingarden et al.^78^ conducted a stability analysis on the post-FMT patient samples and determined that the variation seen in the study’s recipients was akin to normal variations in gut microbiomes. This suggests that the recipients maintained a higher community richness that was induced by the FMT, but that their microbiome was again “personalized,” possibly resulting from participants’ unique environmental exposures.^34^ On the other hand, some studies reported significant similarities to the donated microbiomes 1 to 4.5 years after FMT intervention.^79,113^ While not always possible or practical due to financial, personnel, or time constraints, long-term follow-up in FMT studies can highlight positive outcomes and can support a better understanding of FMT impacts on the human gut microbiome.

### Longitudinal statistics

The majority of FMT studies collect microbiome samples longitudinally. Repeated measures over time introduce data analysis challenges, including dependent data, irregularly timed data, missing timepoints, and dropped subjects.^114^

**Figure 3.**
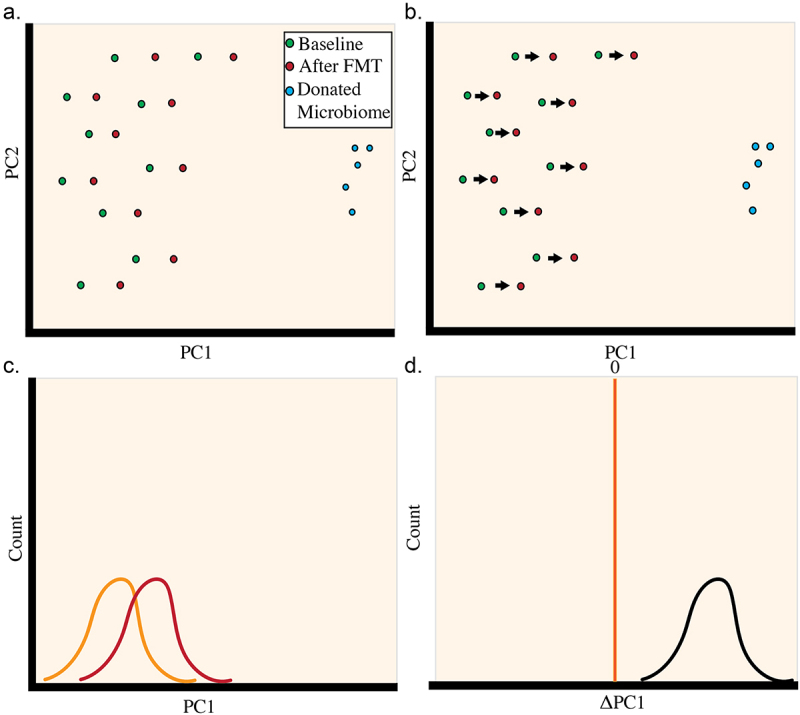
First distances capture rate of change. a) Baseline and after FMT groups do not look like distinct microbiomes. b) Looking at change on a per individual basis however captures that each subject shifted to toward the donated microbiome on the PC axis 1. c) Distributions of baseline and after FMT samples, as described by PC1, overlap, suggesting no significant difference. D) the mean of the distribution of change between baseline and after FMT samples as described by PC1 differs from zero, suggesting a significant directional change in the microbiome composition with treatment. This figure is created as an example and contains simulated data. *this figure was adapted from ASM microbe volume 9, number 10, 2014 with permission from ASM.*

One of the primary challenges in analyzing longitudinal data is the dependence of data samples within a subject. Many statistical tests assume independent data points and repeated measures from the same subject violate this assumption, thus limiting the statistical tests that can be applied. Further, comparing timepoints to each other without pairing samples from the same subject can reduce statistical power for assessing the impact of an FMT or other microbiome intervention. For example, Figure 3(a) shows that the groups “Baseline” and “Post-FMT” might not be statistically different based on their clustering together visually and having largely overlapping distributions (Figure 3(c)). However, if change between the adjacent timepoints is calculated on a per-subject basis (i.e., a paired-difference assessment) directionality of change can be detected (Figure 3(b)), and the mean of that distribution could be tested for statistically significant difference from zero (Figure 3(d)). Thus, applying the appropriate statistical test can highlight important outcomes that otherwise would be obscured.^115^ Another technique for analyzing longitudinal data that was common in the reviewed studies was using linear modeling techniques that support tracking change over time and correlations with other variables.^116^ Appropriately constructed models can control the effects of correlated data or irregular time points.^11,114^

### Guidelines for sample collection to facilitate resilience investigation

Sample collection strategies and timing varied widely in the studies reviewed here, including which types of samples were collected, frequency of sampling, and duration of sampling. Here, we provide guidelines for sampling to best enable assessment of engraftment. When working with human subjects, particularly those who are already burdened with illness, achieving these ideals may not always be feasible.

At a bare minimum, to assess microbial change with FMT, samples must be collected before and after the FMT. We also consider it critical to sample the donated microbiome at time of donation. With these three samples per subject, it is possible to track *Community Coalescence*, and the transfer of *Indicator Features*. (If a recipient receives multiple doses of FMT from different sources (as in Kang et al.^33^ each of the sources should be sequenced. Integration of antibiotics pretreatment with multiple FMT doses, as applied in Kang et al.,^33^ is sometimes referred to as Microbiota Transplant Therapy, or MTT.) However, these three samples do not provide enough information to assess *Resilience* of the FMT.

Ideally, multiple samples will be collected from the recipient prior to and after the FMT, beginning as soon as possible after FMT intervention (e.g., first bowel movement following FMT) so that low engraftment extent at the beginning of the procedure could be identified and potentially rectified. Multiple pre-FMT timepoints provide a more representative characterization of the recipient’s microbiome by allowing for the averaging of normal temporal variation. Additionally, as pre-FMT treatments such as antibiotics are often administered, tracking microbiome changes across these treatments enables a more precise determination of whether observed shifts are attributable to the FMT itself rather than pre-treatment interventions. Multiple timepoints following FMT is necessary for understanding the resilience of the fecal transplant.

It is not currently clear what length of time samples should be collected for following FMT. Collecting and reporting on post-FMT microbiomes will aid in our understanding of what range of resilience should be expected, what range of resilience should be considered abnormal, and whether resilience (or lack thereof) is associated with longer term clinical outcomes.

## Conclusions

In this manuscript, we summarize methods and approaches applied in literature to date for quantifying FMT engraftment in the context of three ecological concepts. The first concept, *Community Coalescence*, provides a whole-microbiome overview of change with FMT intervention. In the FMT literature, it is applied to assess relative similarity between donor and recipient before and after FMT intervention. The three approaches we identified for assessing *Community Coalescence* include alpha diversity, beta diversity, and microbial source tracking. Alpha diversity can describe whether the microbiome richness or evenness changes after FMT but does not show the changes occuring in microbiome composition, and therefore cannot determine how the recipient microbiome was altered by the donated microbiome. Beta diversity assesses whether the microbiome composition of the recipient shifted toward the donated microbiome and/or away from the recipient’s baseline microbiome, based on a community-wide overview. Source tracking methods track the proportion of donated microbiome features that successfully engrafted into the recipient. These metrics provide insight into high-level microbiome change with FMT but do not report changes on the level of specific features.

The second concept, *Indicator Features*, tracks specific feature abundance or presence/absence changes with FMT intervention which can serve as more interpretable evidence for engraftment. However, examining *Indicator Features* does not capture the changes in the microbiome composition as a whole, and can be impacted, for example, by low abundance features falling below levels of detection in either pre- or post-transplant samples. This concept can be explored by analyzing the differential abundance of features before and after FMT, ideally using paired samples or predictive linear models and provides a per-feature overview of engraftment. Features of interest can be either derived from the current study samples or pre-determined independently (e.g., from related literature).

The third concept, *Resilience*, captures whether changes in the recipient’s microbiome following FMT are resistant to reverting back to the baseline microbiome, although the microbiome does not need to maintain similarity to the donated microbiome long term. *Resilience* tends to be investigated using assessments of *Community Coalessence* or *Indicator Feature* tracking applied longitudinally at more than one post-FMT timepoint.

Taken together, these three concepts of FMT engraftment create a framework for evaluating engraftment to assess how the microbiome shifted compositionally, what features were important in that shift and the resilience of the shift. Each on their own does not completely answer the question of whether a microbiome engrafted. Rather, each method provides a different view of microbiome engraftment. We recommend applying approaches that address each of these three concepts, and we expect that this ecological framing of microbiome engraftment extent can facilitate communication about engraftment within and across studies.

### Comparing across studies

Comparing engraftment results across FMT studies is difficult because the different metrics used are generally not directly comparable across studies. For example, alpha diversity metrics such as Faith’s PD and Observed Features are difficult to directly compare because they have different meanings: Faith’s PD includes phylogenetic distances and Observed Features does not. Even comparing the same metric across studies can be difficult because results are impacted by differences in primer choices, depth of sequencing, and sequencing quality control approaches (and phylogenetic metrics are additionally impacted by the approach used for creating the phylogenetic tree). As a result, comparing microbiome engraftment extent across studies tends to be qualitative, focusing on high-level outcomes like “distance to donated microbiome decreased following FMT”. Creating standardized methods to assess engraftment is the first step toward addressing this problem and enabling for more direct comparison across studies that quantify engraftment.

### So, did the microbiome engraft?

Assessing whether a donated microbiome engrafted following FMT, and relating that information to clinical outcomes, remains challenging, in part because the suitable answer to the question of engraftment is not simply “yes” or “no” (Figure 1a): we suggest that a quantitative metric of engraftment extent is more appropriate than a binary measure of engraftment success (Figure 1(b)).

We do not attempt to define an exact threshold for engraftment extent, but aim to describe a framework and approaches for quantifying and communicating engraftment extent. Further, due to a lack of standard methods, the challenges with comparing measures of engraftment extent across studies, and contradictory findings linking success of FMT with clinical outcomes, make it unclear whether crossing (currently undefined) thresholds of engraftment extent is linked with positive clinical outcomes.

Advancing FMT research requires the development of software tools that allow research teams to systematically assess all concepts using standardized approaches, enhancing the interpretation of results. These assessments should ultimately be translated into clinician-targeted reports to support informed decision-making in clinical settings. Toward this end, some of the authors of this manuscript have recently released a QIIME 2 plugin, q2-fmt,^117^ which implements many of the methods discussed here and facilitates the continual exploration of FMT engraftment. Routinely linking engraftment extent, as defined by this framework, to clinical outcomes in FMT research and praxis will help identify the most informative engraftment measures and establish target engraftment levels for optimizing clinical outcomes.

## Supplementary Material

Supplemental Material
