# Supplementary material for: A review of engraftment assessments following fecal microbiota transplant
Source: Gut Microbes. 2025 Jul 2;17(1):2525478. doi: 10.1080/19490976.2025.2525478 (PMC12233830; doi:10.1080/19490976.2025.2525478)
Supplement: Supplemental Material [file KGMI_A_2525478_SM1469.zip › Supplemental_Text_1.docx]

Supplemental Text 1

In this supplemental text we present methods for comparing alpha diversity in the context of FMT and common approaches for visualizing FMT engraftment assessments with schematic diagrams (as supplemental figures) using artificial data and real-world examples from the FMT

literature. The visualizations presented here are intended to provide guidance for researchers new to presenting FMT engraftment results.

# Alpha Diversity Comparisons in FMT studies

Alpha diversity estimates within-sample diversity, including community richness and community evenness. Since gut microbiome health is often correlated with higher microbial richness[^1–3^](https://paperpile.com/c/e0VSDx/848XP%2B6FRQc%2BWBBt5), richness is often used as a metric of gut microbiome health, although no clear

causative relationship between the two has been generally established. A variety of metrics are used to assess alpha diversity. These include richness metrics such as Faith’s Phylogenetic Diversity (PD)[^4^](https://paperpile.com/c/e0VSDx/PijEG), Observed Features, Simpson Diversity Score[^5^](https://paperpile.com/c/e0VSDx/anOVD), and Inverse Simpson Diversity Score[^5^](https://paperpile.com/c/e0VSDx/anOVD), Shannon’s Index[^6^](https://paperpile.com/c/e0VSDx/bXPMO), and Pielou’s Evenness.[^7^](https://paperpile.com/c/e0VSDx/nK2a8) To assess FMT engraftment, it is common in the FMT literature to use alpha diversity to compare the recipient to the donated microbiome[^8–13^](https://paperpile.com/c/e0VSDx/UwKPH%2BkpF67%2BjA4B9%2Bn8qTn%2BRKxTJ%2BHqdOY), the recipient after FMT intervention to their pre-treatment baseline[^9,12–20^](https://paperpile.com/c/e0VSDx/5umod%2BTTRWq%2BPQmjz%2BxLrsb%2BY54K3%2BkpF67%2Bx4Clb%2BRKxTJ%2BHqdOY%2BKKpqm), and the recipient to a control.[11,16–18,21–23](https://paperpile.com/c/e0VSDx/PQmjz%2BxLrsb%2BY54K3%2BmMhtj%2BhEeFO%2Bn8qTn%2BcqgTQ)

Alpha diversity metrics generally do not capture what features are shared between the donated microbiome and the recipient, meaning that two individuals can have the same alpha diversity metric value, but have no features in common. For this reason, alpha diversity can’t be used to assess Community Coalescence, though it is sometimes presented that way in FMT literature.

Recipient to Baseline Comparisons

In the reviewed articles, we observed that it was most common to compare the recipient’s gut microbiome richness after FMT to their microbiome richness before FMT. If the donated microbiome’s richness was higher than the recipient’s before FMT (although this information is

not always collected), an increase in community richness after FMT is often taken as a sign that the FMT intervention was effective at altering the microbiome.

Post-FMT alpha diversity was significantly increased relative to baseline alpha diversity in many of the studies we reviewed.[^10,12–19,22,24–28^](https://paperpile.com/c/e0VSDx/5umod%2BTTRWq%2BPQmjz%2BxLrsb%2BY54K3%2BjA4B9%2Bx4Clb%2BhEeFO%2BRKxTJ%2BHqdOY%2BctqMa%2BUVuLd%2B6wwl4%2BXeD95%2BSdAIb) Some studies identified no statistically significant change in alpha diversity[^29,30^](https://paperpile.com/c/e0VSDx/Yf202%2BcMmLo), while others found an apparent increase over time (in

some cases statistically significant, but in other cases no statistical test was applied).[^31–34^](https://paperpile.com/c/e0VSDx/hxozz%2BLYWAK%2BEEW3g%2BXMh7k) Even though most studies show an increase in alpha diversity following transplant, some studies did present a decrease in alpha diversity following transplant.[^35,36^](https://paperpile.com/c/e0VSDx/H11jJ%2Bwpy9j) For example, Su et al. (2022)[^35^](https://paperpile.com/c/e0VSDx/H11jJ) tracked community richness of their recipients over the course of their 90 day study and found a significant decrease in evenness as measured by Pielou’s Evenness but no significant difference in richness as measured by Observed Features following FMT, which is atypical with a “healthy” donated microbiome. This might be because of the specific diet that was given after the FMT intervention, as the diet-only cohort (who did not receive an FMT) saw an even more drastic

decrease in alpha diversity than the FMT group.

Sometimes an individual’s baseline sample is already taken after antibiotic use or other treatments, like allo-HCT, which are known to reduce the richness of the gut microbiome.

Baseline samples are ideally taken multiple times, including before antibiotics or other

treatments [^15–17,31–33,35^](https://paperpile.com/c/e0VSDx/H11jJ%2BTTRWq%2BPQmjz%2BxLrsb%2Bhxozz%2BLYWAK%2BEEW3g) , but that is not always practical in human subjects research. Amorim et al. (2022)[^19^](https://paperpile.com/c/e0VSDx/x4Clb) did not measure their baseline prior to antibiotic intervention, and in post-FMT samples found an increase in Shannon’s Diversity and Observed Species relative to pre-FMT intervention. In a case like this, it is not possible to determine if an increase in richness is a result of recovery from antibiotic use, the FMT intervention, or a combination of the two. In another

example of comparing to a baseline sample after treatment, DeFilipp et al. (2018)[^18^](https://paperpile.com/c/e0VSDx/Y54K3) showed increased richness by collecting a separate baseline after allo-HCT, using Inverse Simpson Diversity Score to track community richness after FMT intervention relative to two timepoints: before and after allo-HCT treatment.

Recipient to Donated Microbiome Comparisons

Another common approach for assessing engraftment with alpha diversity is comparing the donated microbiome to the recipient’s gut microbiome throughout the FMT study. The

purpose of this approach is to track whether the alpha diversity of the recipient is becoming more similar to the donated microbiome over the course of the study.

Some studies showed a significant difference at baseline between the donated

microbiome and the recipients’ microbiome, and then observed recipient community richness

become more similar to that of the donated microbiome after FMT.[^8–10,24,37,38^](https://paperpile.com/c/e0VSDx/UwKPH%2BkpF67%2BjA4B9%2BIkVUJ%2BuE9o6%2BctqMa) Interestingly, Hazan et al. (2021)[^9^](https://paperpile.com/c/e0VSDx/kpF67) used Simpson’s and Shannon’s diversity indexes and noted that their single patient who did not experience microbiome engraftment had a relatively high community richness prior to FMT intervention, but decreased after FMT intervention. Davar et al. (2021)[^39^](https://paperpile.com/c/e0VSDx/rpNN) found no significant differences between the donated microbiome and baseline recipient samples, so they did not use change in alpha diversity as a metric for assessing engraftment.

Recipient to Control Comparisons

The last common comparison type that we observed involved comparing FMT recipients to a control group that did not receive an FMT, to assess the impact of the intervention.[^21,22,37^](https://paperpile.com/c/e0VSDx/mMhtj%2BIkVUJ%2BhEeFO) For example, Kong et al. (2020)[^21^](https://paperpile.com/c/e0VSDx/mMhtj) used species level change in Shannon’s index of their FMT

participants compared to their Sham FMT participants (patients who received a placebo). Ma et al. (2023)[^37^](https://paperpile.com/c/e0VSDx/IkVUJ) also compared their findings to a group that was only given PBS. Similarly, Wang et al. (2020)[^40^](https://paperpile.com/c/e0VSDx/LnR1T) compared FMT recipients at different timepoints to their control group, who received no antibiotics or FMT. Comparisons to controls can elucidate how FMT pre-treatments, like antibiotics, might affect the microbiome[^11^](https://paperpile.com/c/e0VSDx/n8qTn). Wang et al. (2020)[^40^](https://paperpile.com/c/e0VSDx/LnR1T) compared FMT recipients to their spontaneous recovery group, which received antibiotics but no FMT. Another study compared individuals who received allo-HCT and FMT to individuals that received solely

allo-HCT treatment.[^18^](https://paperpile.com/c/e0VSDx/Y54K3) Finally, Kang et al. (2017)[^16^](https://paperpile.com/c/e0VSDx/PQmjz) and Kang et al. (2019)[^17^](https://paperpile.com/c/e0VSDx/xLrsb) compared Faith’s PD in transplant recipients to an age and gender matched control group.

# Alpha Diversity Visualizations

Alpha diversity metrics are commonly visualized using a boxplot with a scatter plot overlaid to show the variation in diversity of the subjects before and after their FMT (Supplemental

Figure 1 ).[^16,17,33–35,41^](https://paperpile.com/c/e0VSDx/H11jJ%2BPQmjz%2BxLrsb%2BEdaks%2BEEW3g%2BXMh7k) This helps visualize variation of community richness. Similarly, DeFilipp et al. (2018)[^18^](https://paperpile.com/c/e0VSDx/Y54K3) used a scatter plot with a median line, and Singh et al. used only a scatter plot.[^14^](https://paperpile.com/c/e0VSDx/5umod) Other studies used a trendline, which illustrates how community richness shifts throughout the

course of the study (Supplemental Figure 2C).[^19,21,22,31,36^](https://paperpile.com/c/e0VSDx/hxozz%2Bx4Clb%2BmMhtj%2BhEeFO%2Bwpy9j) Some researchers incorporate a median line illustrating donated microbiome alpha diversity to help illustrate the recipients’ alpha diversity compared to the donated microbiomes’ (Supplemental Figure 2A).[^16,17,31^](https://paperpile.com/c/e0VSDx/PQmjz%2BxLrsb%2Bhxozz)

# Beta Diversity Visualizations

The most common way to visualize beta diversity in the microbiome literature, and in the studies we reviewed, is through ordination with a method such as Principal Coordinates Analysis (PCoA), and subsequent viewing of the first two or three PCoA axes in a scatter plot (Supplemental Figure 4A).[^8,15,19,22,34,35,39,41–45^](https://paperpile.com/c/e0VSDx/H11jJ%2BUpZO%2BrpNN%2BD9yFk%2BTTRWq%2BEdaks%2BUwKPH%2Bx4Clb%2BhEeFO%2BXMh7k%2BFSTRt%2B3LSHQ) PCoA plots[^46^](https://paperpile.com/c/e0VSDx/LLA0p) are convenient approaches for illustrating similarities and differences between groups of samples based on beta diversity distances, frequently highlighting whether groups of samples cluster together (indicating similarity in microbiome composition) by using group-specific sample colors or shapes. In the reviewed studies, sample groups were treatment groups and/or time points. Two studies plotted

the distribution of samples along PCoA axis 1 as boxplots (Supplemental Figure 4D)[^35,39^](https://paperpile.com/c/e0VSDx/H11jJ%2BrpNN). PCoA axis values could also be presented across time in an FMT study using a plot like that presented in Supplemental Figure 3 (replacing alpha diversity values with the values of specific samples along a single PCoA axis).

While PCoA plots help with visualizing differences in beta diversity on a broad scale, comparing the underlying distances themselves removes a layer of dimensionality reduction. Box plots, typically with jitter plots overlaid, are commonly used to visualize recipients’ distances to donated microbiomes (Supplemental Figure 3A), their baseline samples (Supplemental Figure 3B, Supplemental Figure 4B), or to control group samples. Multiple box plots can be displayed next to each other to illustrate changes over time or across treatment groups (Supplemental

Figure 3).[^16,17,19,22^](https://paperpile.com/c/e0VSDx/PQmjz%2BxLrsb%2Bx4Clb%2BhEeFO) This allows readers to see the variation of distances to donated microbiomes within groups. As illustrated in Supplemental Figure 3, distance to the donated microbiome often continues to decrease in the weeks after treatment, suggesting open questions about the

microbiome engraftment process and highlighting that summaries such as these do not exclusively present engraftment extent[^47^](https://paperpile.com/c/e0VSDx/zgPLD). Another way to visualize distance to donated

microbiome is with trendlines tracking recipients’ distance from or similarity to a donated microbiome over time, which helps illustrates recipients’ individual microbiome shifts (Supplemental Figure 4C).[^21,33,44^](https://paperpile.com/c/e0VSDx/mMhtj%2BEEW3g%2BFSTRt)

# Source Tracking Visualizations

The most common source tracking visualizations are trendlines (Supplemental Figure 6B)[^8,10,31,48^](https://paperpile.com/c/e0VSDx/hxozz%2BUwKPH%2BjA4B9%2BmHqlq), and barplots (Supplemental Figure 5A, Supplemental Figure 6A).[^18,34,41,48^](https://paperpile.com/c/e0VSDx/Edaks%2BY54K3%2BXMh7k%2BmHqlq) Trendlines are typically used to understand how the proportions of microbial sources change in the recipient’s microbiome over time, while barplots are typically used to understand how specific recipients or groups are changing with respect to microbial source across time (Supplemental

Figure 5A). Scatter plots[^14^](https://paperpile.com/c/e0VSDx/5umod) and boxplots[^34^](https://paperpile.com/c/e0VSDx/XMh7k) are also used, but they are not as common. Alternatively, a heatmap might be able to capture a subject's PEDS value over time, but only one study that we reviewed used a heatmap to visualize source tracking (Supplemental Figure 5A).[^13^](https://paperpile.com/c/e0VSDx/HqdOY)

# Features Abundance Tracking Visualizations

Differential abundance can be complex to visualize because most differential abundance methods that are relevant to microbiome data transform the data for testing. This means that

teams have to visualize using either transformed data (Supplemental Figure 7A), which can be hard to interpret, or relative abundance (Supplemental Figure 7B), which is easier to interpret but may not clearly reflect transformations made to the data to support the test. Boxplots were one of the most common visualizations in the discussed studies (Supplemental Figure 8C).[^16,17,32,34,44^](https://paperpile.com/c/e0VSDx/PQmjz%2BxLrsb%2BLYWAK%2BXMh7k%2BFSTRt) Similarly, Parker et al. (2022)[^15^](https://paperpile.com/c/e0VSDx/TTRWq) used a diverging barplot with mean differences in centered log ratio and standard error (Supplemental Figure 8A).[^15^](https://paperpile.com/c/e0VSDx/TTRWq) Another common visualization is a heat map of relative abundance (Supplemental Figure 8B)[^34^](https://paperpile.com/c/e0VSDx/XMh7k), or normalized distance.[^21^](https://paperpile.com/c/e0VSDx/mMhtj) Trendlines are also used to track relative abundance of the differentially abundant species over time (Supplemental Figure 7B)[^31,44^](https://paperpile.com/c/e0VSDx/hxozz%2BFSTRt), while Baruch et al. (2021) and Defillip et al. (2018) use scatter plots.[^18,42^](https://paperpile.com/c/e0VSDx/UpZO%2BY54K3)

# Supplemental Figures


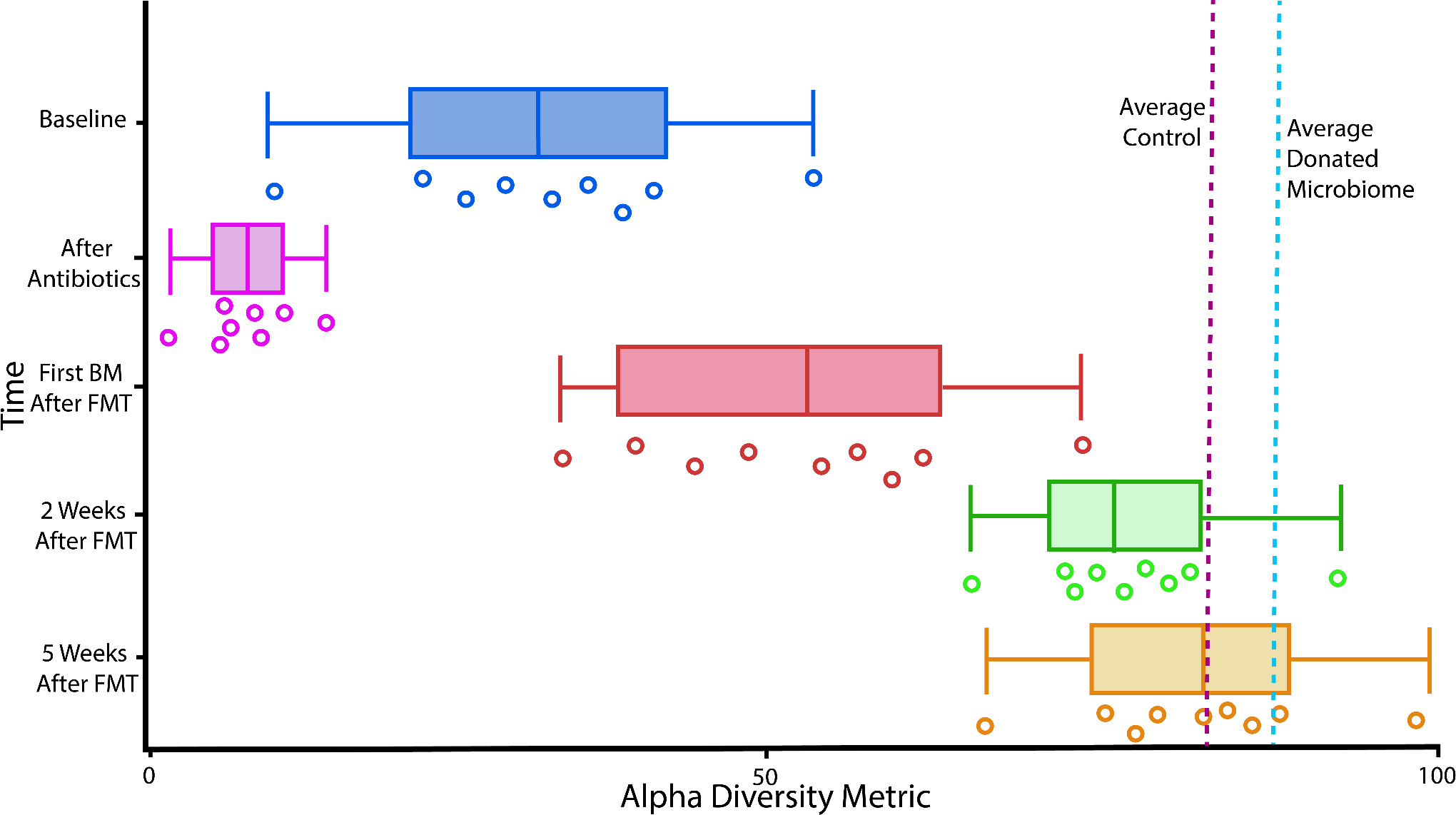


**Supplemental Figure 1: Schematic of Chimeric Asymmetric Community Coalescence assessment with an alpha diversity metric.** This visualization summarizes alpha diversity distributions at each time point with box plots, provides additional detail on the distributions in the corresponding jitter plots, and the *Average Control* and *Average Donated Microbiome* reference lines aid in contextualizing the metric. With appropriate statistics, such as the Wilcoxon signed rank test[^49^](https://paperpile.com/c/e0VSDx/rgyby), this plot could be used to suggest engraftment in recipients as assessed by Chimeric Asymmetric Community Coalescence with an alpha diversity metric, but we note again the caveat that similar measures of alpha diversity could be achieved with no shared features, so we do not consider this to be strong evidence of engraftment. *First BM After FMT Treatment* indicates the recipient's first bowel movement (BM) following the fecal microbiota transplant. This data is fake and is meant to illustrate how to show alpha diversity as a feature of engraftment extent.


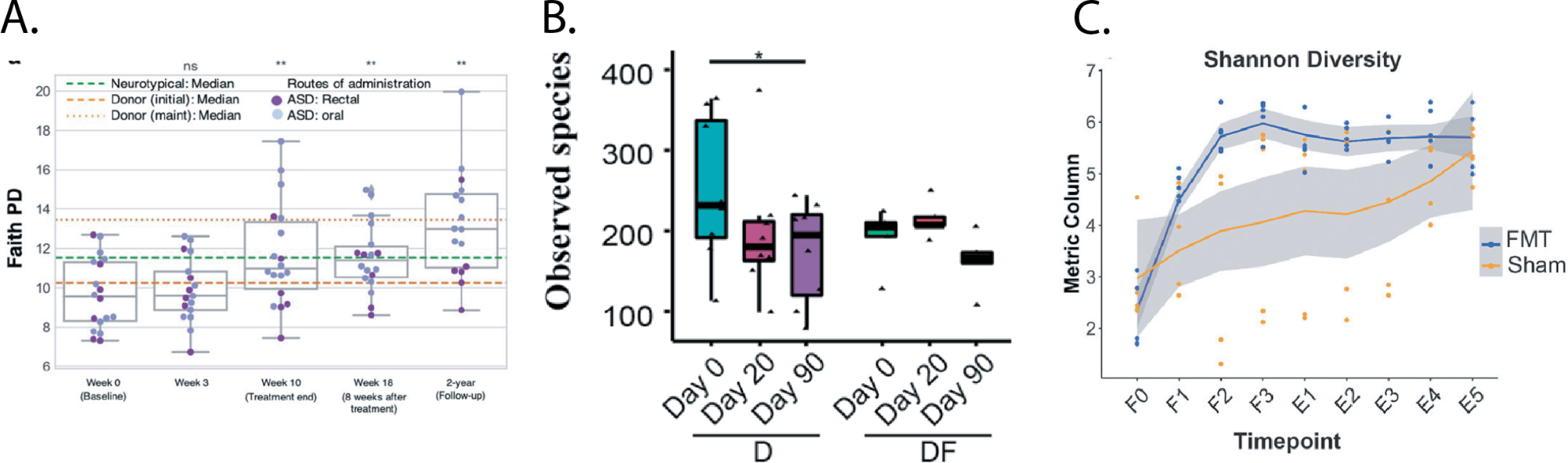


**Supplemental Figure 2: Examples of Common Alpha Diversity Visualizations.** A) Kang et al. 2019 B) Su et al. 2022 C) Amorim et al. 2022. These figures have been reproduced in accordance with their Creative Commons licenses.


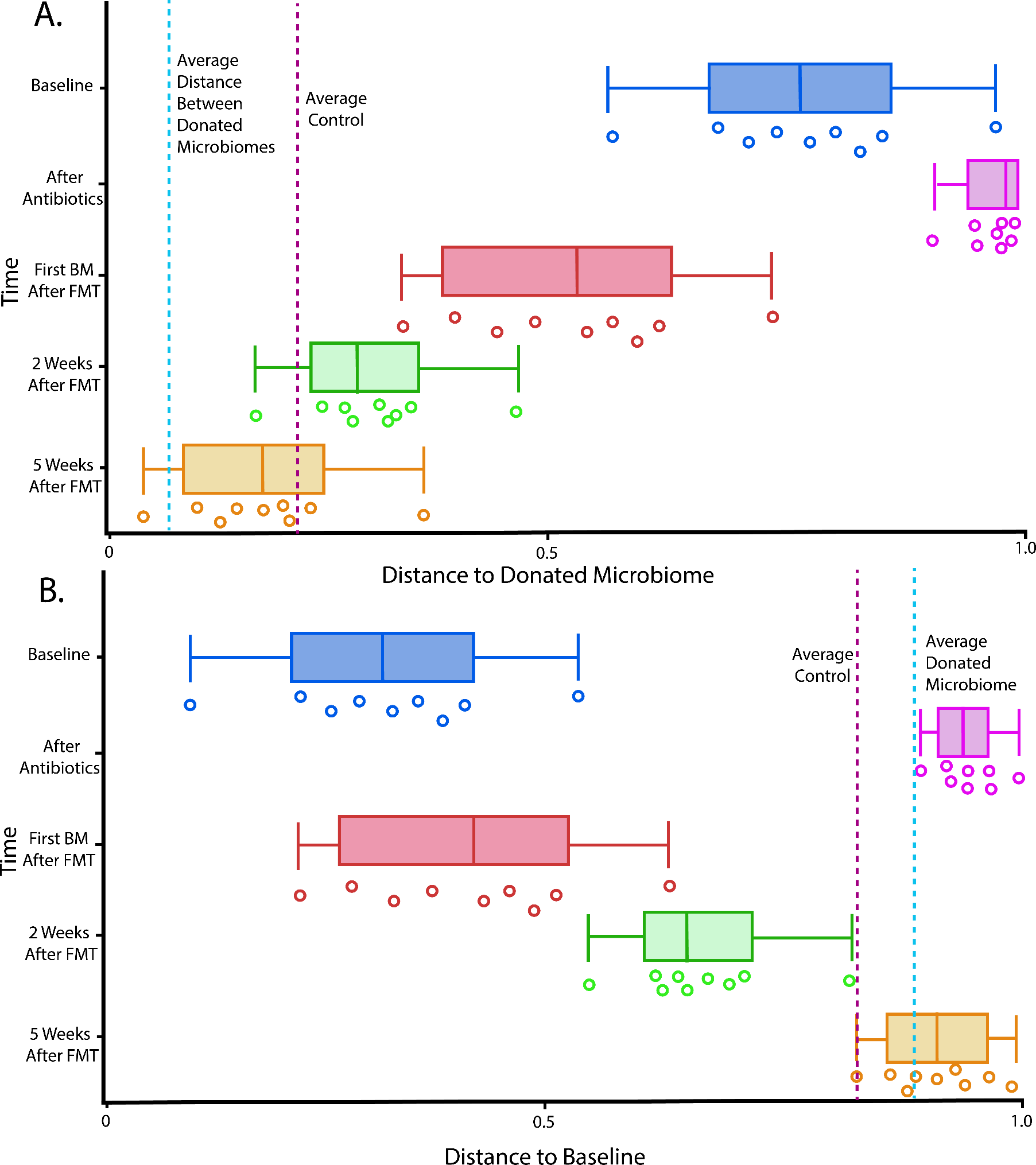


**Supplemental Figure 3: Schematic of *Chimeric Asymmetric Community Coalescence* assessment with beta diversity distances between samples**. This visualization illustrates distances to (A) donated microbiomes and (B) individuals' baseline samples. Relevant average control and donor distance reference lines aid in contextualizing distances. With appropriate statistics, such as Wilcoxon signed rank test, panel A could be used to suggest engraftment based on a decreasing distance to the donated microbiome samples with treatment and panel B could be used to suggest engraftment based on increasing distance from baseline samples with treatment. *First BM After FMT Treatment* indicates the recipient's first bowel movement (BM) following the fecal microbiota transplant. The data presented here is fake and is meant to illustrate how to use beta diversity to highlight engraftment.


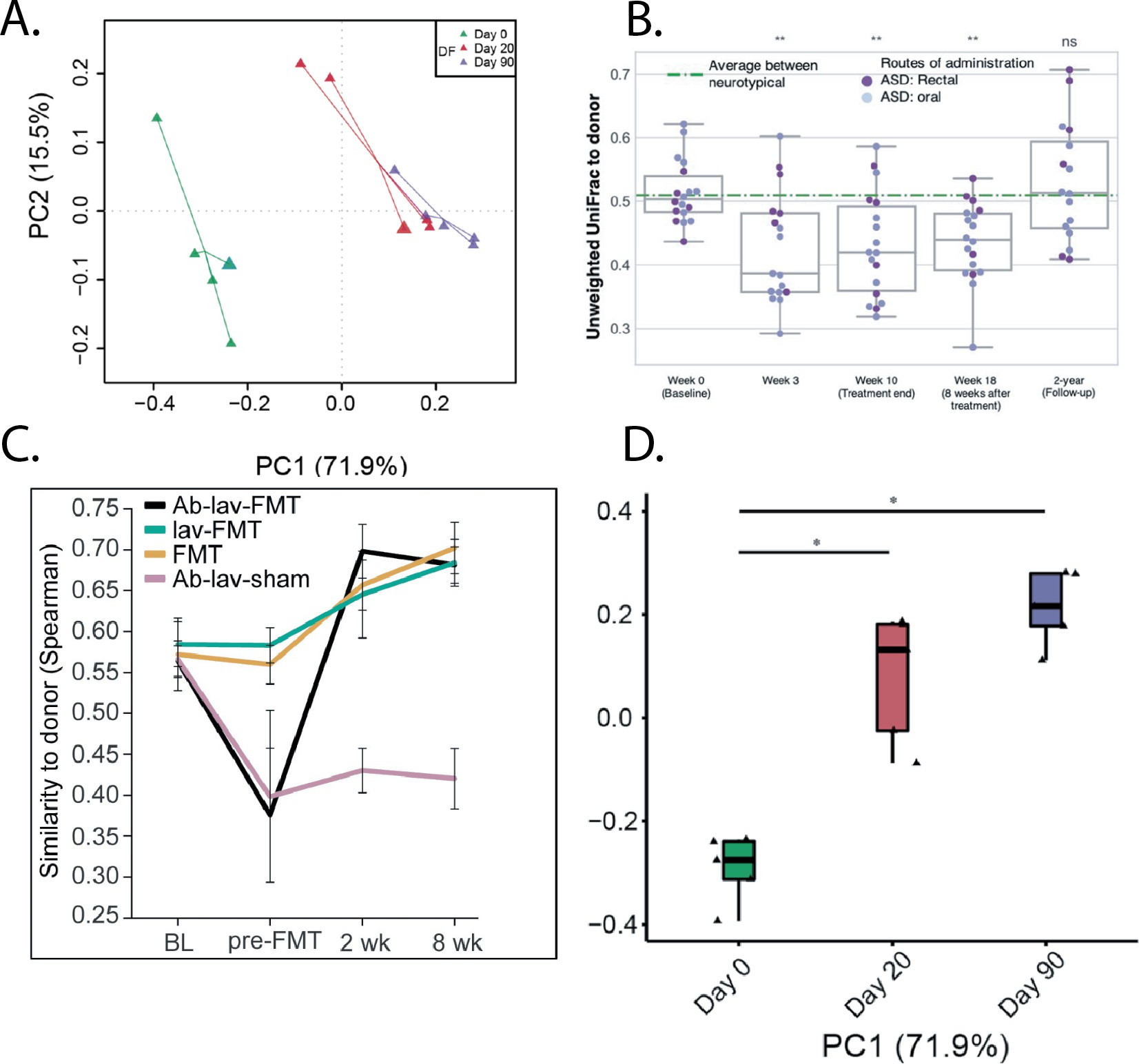


**Supplemental Figure 4: Examples of Common Beta Diversity Visualizations.** A) Su et al. 2022 , B) Kang et al. 2019, C) Freitag et al. 2019, D) Su et al. 2022. These figures have been reproduced in accordance with their Creative Commons licenses.


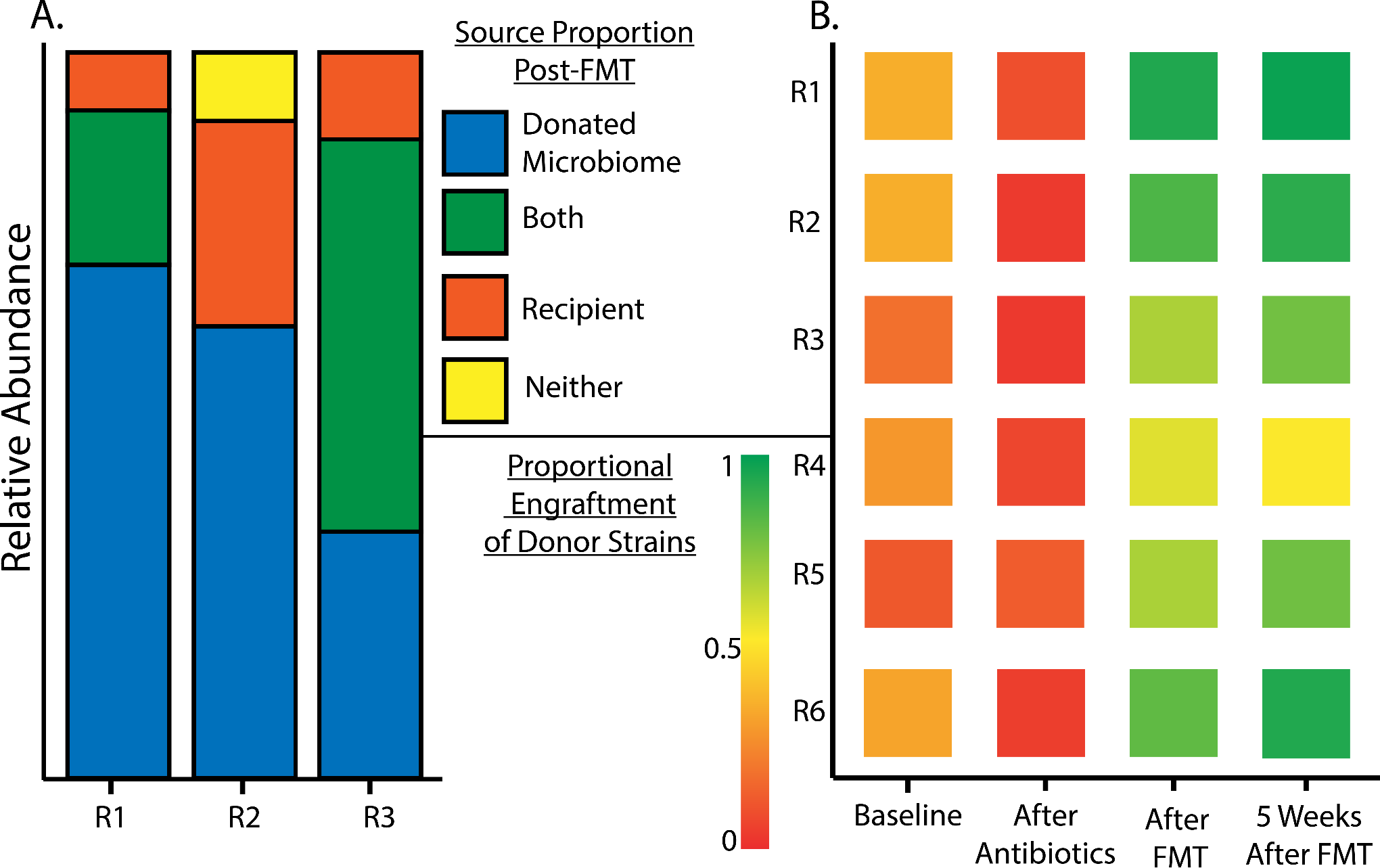


**Supplemental Figure 5: Schematic of *Chimeric Asymmetric Community Coalescence* assessment with microbiome feature source tracking.** (A) Recipient microbiomes are described at a single post-FMT timepoint in terms of the source of each feature in the microbiome: the donated microbiome, the recipient’s baseline microbiome (recipient), both, or neither. This could be generalized to provide this information across multiple timepoints as well.

(B) PEDS is tracked across time and across subjects, illustrating larger values post-FMT relative to baseline. R1 - R6 refer to 6 theoretical recipients of an FMT. The data presented here is fake and is meant to illustrate how to use source tracking to highlight engraftment.


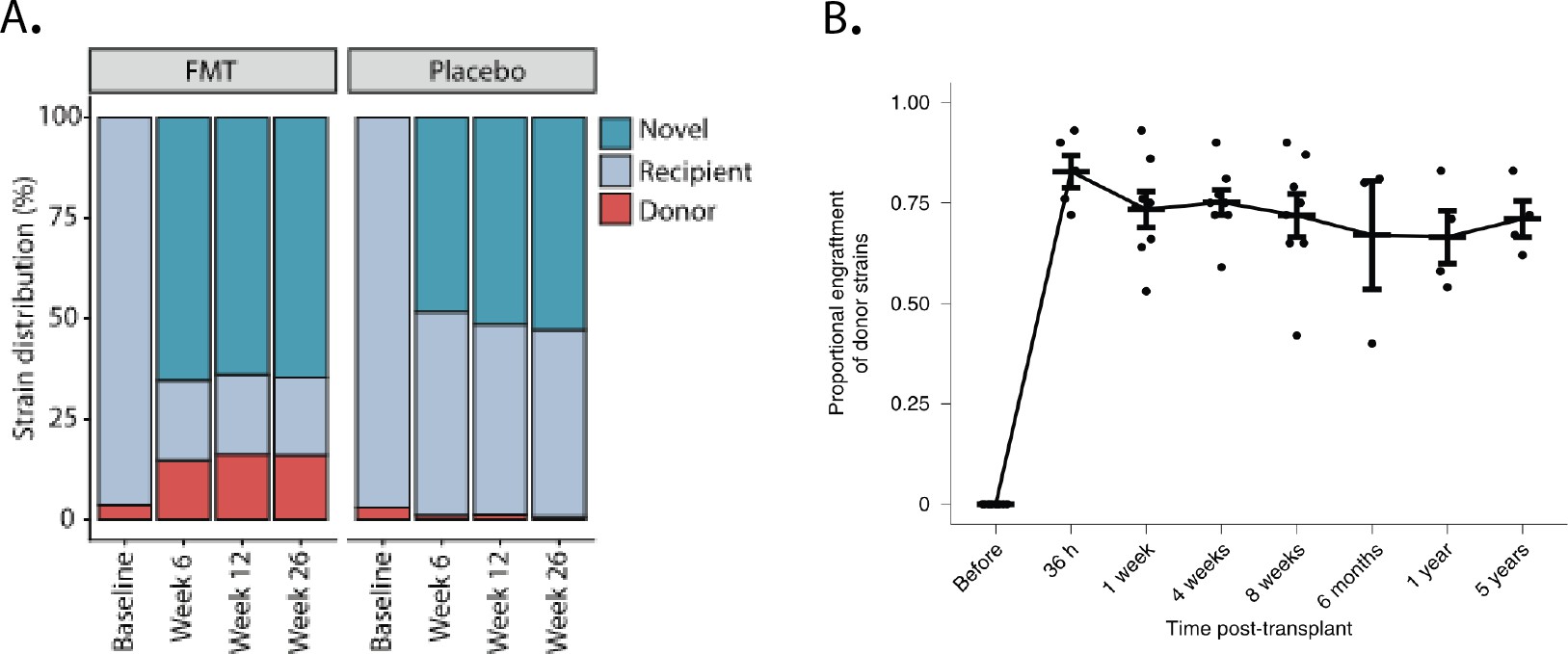


**Supplemental Figure 6: Examples of Common FMT Source Tracking Visualizations.** A) Wilson et al. 2021, B) Aggarwala et al. 2021. These figures have been reproduced in accordance with their Creative Commons licenses.


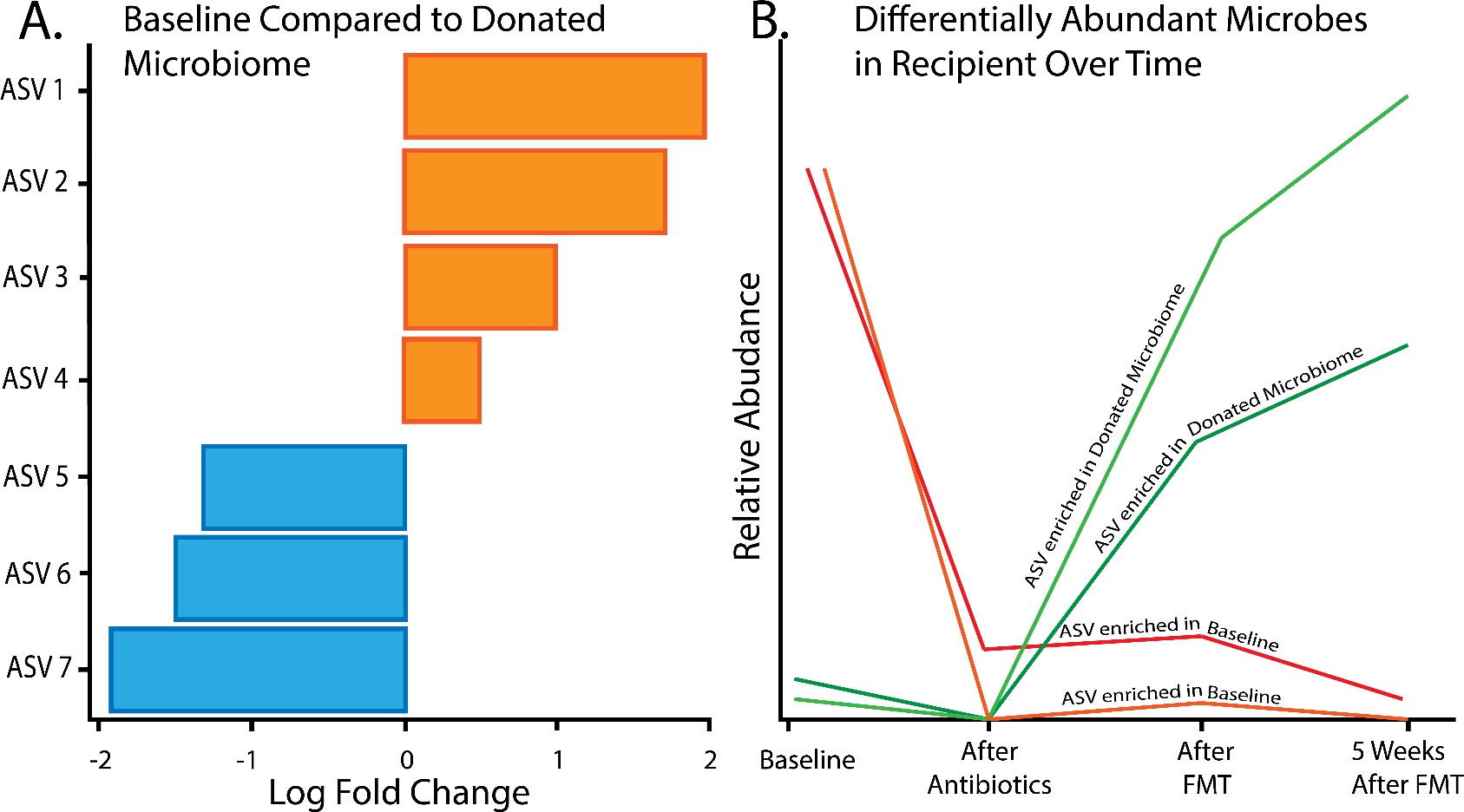


**Supplemental Figure 7: Feature Abundance Tracking Visualization.** This visualization contains **(A)** diverging bar plot with log fold change to illustrate differentially abundant microbes between the donated microbiome and baseline. Orange bars indicate ASVs enriched in the baseline compared to donated microbiomes, while blue bars indicate ASVs that are depleted in the baseline compared to donated microbiomes. Subsection **(B)** if the visualization tracks how the relative abundance changes over time. The data presented here is generated and is meant to illustrate how to use feature tracking to highlight engraftment.


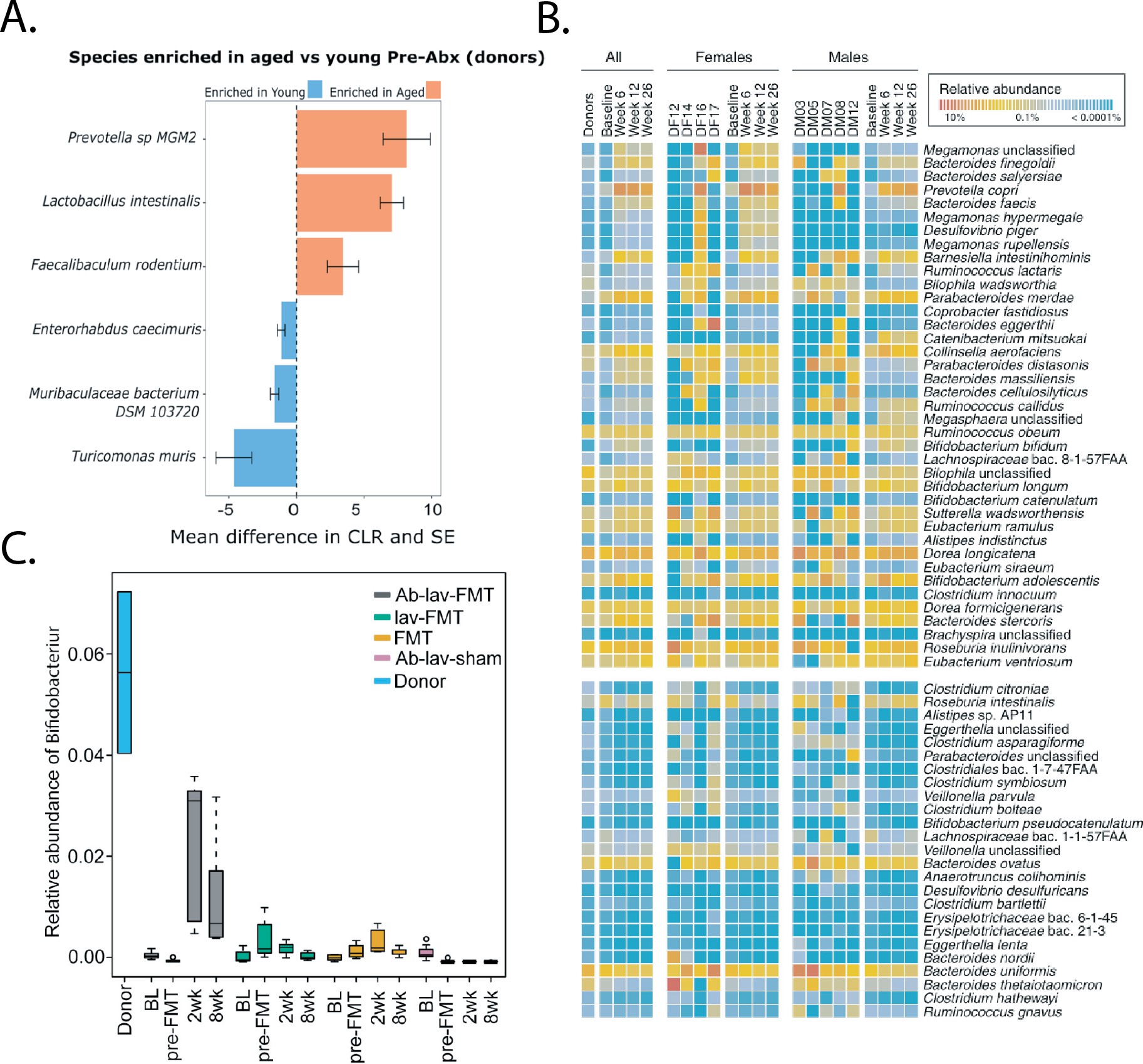


**Supplemental Figure 8: Examples of Common Feature Abundance Visualizations.** A) Parker et al. 2022, B) Wilson et al. 2021, C) Freitag et al. 2019. These figures have been reproduced in accordance with their Creative Commons licenses.

# Supplemental Works Cited

1. [Lozupone, C. A., Stombaugh, J. I., Gordon, J. I., Jansson, J. K. & Knight, R. Diversity,](http://paperpile.com/b/e0VSDx/848XP) [stability and resilience of the human gut microbiota. *Nature* **489**, 220–230 (2012).](http://paperpile.com/b/e0VSDx/848XP)
2. [Mosca, A., Leclerc, M. & Hugot, J. P. Gut Microbiota Diversity and Human Diseases:](http://paperpile.com/b/e0VSDx/6FRQc) [Should We Reintroduce Key Predators in Our Ecosystem? *Front. Microbiol.* **7**, (2016).](http://paperpile.com/b/e0VSDx/6FRQc)
3. [Scepanovic, P. *et al.* A comprehensive assessment of demographic, environmental, and](http://paperpile.com/b/e0VSDx/WBBt5) [host genetic associations with gut microbiome diversity in healthy individuals. *Microbiome*](http://paperpile.com/b/e0VSDx/WBBt5) [**7**, 130 (2019).](http://paperpile.com/b/e0VSDx/WBBt5)
4. [Faith, D. P. Conservation evaluation and phylogenetic diversity. *Biol. Conserv.* **61**, 1–10](http://paperpile.com/b/e0VSDx/PijEG) [(1992).](http://paperpile.com/b/e0VSDx/PijEG)
5. [Simpson, E. H. Measurement of Diversity. *Nature* **163**, 688–688 (1949).](http://paperpile.com/b/e0VSDx/anOVD)
6. [Shannon, C. E. A mathematical theory of communication. *The Bell System Technical*](http://paperpile.com/b/e0VSDx/bXPMO) [*Journal* **27**, 379–423 (1948).](http://paperpile.com/b/e0VSDx/bXPMO)
7. [Pielou, E. C. The measurement of diversity in different types of biological collections. *J.*](http://paperpile.com/b/e0VSDx/nK2a8) [*Theor. Biol.* **13**, 131–144 (1966).](http://paperpile.com/b/e0VSDx/nK2a8)
8. [Gopalakrishnan, V. *et al.* Engraftment of Bacteria after Fecal Microbiota Transplantation Is](http://paperpile.com/b/e0VSDx/UwKPH) [Dependent on Both Frequency of Dosing and Duration of Preparative Antibiotic Regimen.](http://paperpile.com/b/e0VSDx/UwKPH) [*Microorganisms* **9**, 1399 (2021).](http://paperpile.com/b/e0VSDx/UwKPH)
9. [Hazan, S., Dave, S., Papoutsis, A. J., Barrows, B. D. & Borody, T. J. Successful Bacterial](http://paperpile.com/b/e0VSDx/kpF67) [Engraftment Identified by Next-Generation Sequencing Predicts Success of Fecal](http://paperpile.com/b/e0VSDx/kpF67) [Microbiota Transplant for Clostridioides difficile. *Gastroenterol. Res. Pract.* **14**, 304–309](http://paperpile.com/b/e0VSDx/kpF67) [(2021).](http://paperpile.com/b/e0VSDx/kpF67)
10. [Staley, C. *et al.* Durable Long-Term Bacterial Engraftment following Encapsulated Fecal](http://paperpile.com/b/e0VSDx/jA4B9) [Microbiota Transplantation To Treat Clostridium difficile Infection. *MBio* **10**, e01586–19](http://paperpile.com/b/e0VSDx/jA4B9) [(2019).](http://paperpile.com/b/e0VSDx/jA4B9)
11. [Zeng, X. *et al.* Fecal microbiota transplantation from young mice rejuvenates aged](http://paperpile.com/b/e0VSDx/n8qTn) [hematopoietic stem cells by suppressing inflammation. *Blood* **141**, 1691–1707 (2023).](http://paperpile.com/b/e0VSDx/n8qTn)
12. [Wu, Z. *et al.* Fecal microbiota transplantation reverses insulin resistance in type 2 diabetes:](http://paperpile.com/b/e0VSDx/RKxTJ) [A randomized, controlled, prospective study. *Front. Cell. Infect. Microbiol.* **12**, 1089991](http://paperpile.com/b/e0VSDx/RKxTJ) [(2022).](http://paperpile.com/b/e0VSDx/RKxTJ)
13. [Routy, B. *et al.* Fecal microbiota transplantation plus anti-PD-1 immunotherapy in advanced](http://paperpile.com/b/e0VSDx/HqdOY) [melanoma: a phase I trial. *Nat. Med.* **29**, 2121–2132 (2023).](http://paperpile.com/b/e0VSDx/HqdOY)
14. [Singh, P. *et al.* Effect of antibiotic pretreatment on bacterial engraftment after Fecal](http://paperpile.com/b/e0VSDx/5umod) [Microbiota Transplant (FMT) in IBS-D. *Gut Microbes* **14**, 2020067.](http://paperpile.com/b/e0VSDx/5umod)
15. [Parker, A. Fecal microbiota transfer between young and aged mice reverses hallmarks of](http://paperpile.com/b/e0VSDx/TTRWq) [the aging gut, eye, and brain. 25 (2022).](http://paperpile.com/b/e0VSDx/TTRWq)
16. [Kang, D.-W. *et al.* Microbiota Transfer Therapy alters gut ecosystem and improves](http://paperpile.com/b/e0VSDx/PQmjz) [gastrointestinal and autism symptoms: an open-label study. *Microbiome* **5**, 10 (2017).](http://paperpile.com/b/e0VSDx/PQmjz)
17. [Kang, D.-W. *et al.* Long-term benefit of Microbiota Transfer Therapy on autism symptoms](http://paperpile.com/b/e0VSDx/xLrsb) [and gut microbiota. *Sci. Rep.* **9**, 5821 (2019).](http://paperpile.com/b/e0VSDx/xLrsb)
18. [DeFilipp, Z. *et al.* Third-party fecal microbiota transplantation following allo-HCT](http://paperpile.com/b/e0VSDx/Y54K3) [reconstitutes microbiome diversity. *Blood Advances* **2**, 745–753 (2018).](http://paperpile.com/b/e0VSDx/Y54K3)
19. [Amorim, N. *et al.* Refining a Protocol for Faecal Microbiota Engraftment in Animal Models](http://paperpile.com/b/e0VSDx/x4Clb) [After Successful Antibiotic-Induced Gut Decontamination. *Frontiers in Medicine* **9**, (2022).](http://paperpile.com/b/e0VSDx/x4Clb)
20. [Paramsothy, S. *et al.* Specific Bacteria and Metabolites Associated With Response to Fecal](http://paperpile.com/b/e0VSDx/KKpqm) [Microbiota Transplantation in Patients With Ulcerative Colitis. *Gastroenterology* **156**,](http://paperpile.com/b/e0VSDx/KKpqm)

[1440–1454.e2 (2019).](http://paperpile.com/b/e0VSDx/KKpqm)

1. [Kong, L. *et al.* Linking strain engraftment in fecal microbiota transplantation with](http://paperpile.com/b/e0VSDx/mMhtj) [maintenance of remission in Crohn’s disease. *Gastroenterology* **159**, 2193–2202.e5 (2020).](http://paperpile.com/b/e0VSDx/mMhtj)
2. [Wang, Y. *et al.* Establishment and resilience of transplanted gut microbiota in aged mice.](http://paperpile.com/b/e0VSDx/hEeFO)

[*iScience* **25**, 103654 (2022).](http://paperpile.com/b/e0VSDx/hEeFO)

1. [Yu, H. *et al.* Fecal microbiota transplantation inhibits colorectal cancer progression:](http://paperpile.com/b/e0VSDx/cqgTQ) [Reversing intestinal microbial dysbiosis to enhance anti-cancer immune responses. *Front.*](http://paperpile.com/b/e0VSDx/cqgTQ) [*Microbiol.* **14**, 1126808 (2023).](http://paperpile.com/b/e0VSDx/cqgTQ)
2. [Staley, C., Kelly, C. R., Brandt, L. J., Khoruts, A. & Sadowsky, M. J. Complete Microbiota](http://paperpile.com/b/e0VSDx/ctqMa) [engraftment is not essential for recovery from recurrent Clostridium difficile infection](http://paperpile.com/b/e0VSDx/ctqMa) [following fecal Microbiota transplantation. *MBio* **7**, (2016).](http://paperpile.com/b/e0VSDx/ctqMa)
3. [Hirten, R. P. *et al.* Microbial engraftment and efficacy of fecal Microbiota transplant for](http://paperpile.com/b/e0VSDx/UVuLd) [Clostridium difficile in patients with and without inflammatory bowel disease. *Inflamm.*](http://paperpile.com/b/e0VSDx/UVuLd) [*Bowel Dis.* **25**, 969–979 (2019).](http://paperpile.com/b/e0VSDx/UVuLd)
4. [Rossen, N. G. *et al.* Findings from a randomized controlled trial of fecal transplantation for](http://paperpile.com/b/e0VSDx/6wwl4) [patients with ulcerative colitis. *Gastroenterology* **149**, 110–118.e4 (2015).](http://paperpile.com/b/e0VSDx/6wwl4)
5. [Jalanka, J. *et al.* Long-term effects on luminal and mucosal microbiota and commonly](http://paperpile.com/b/e0VSDx/XeD95) [acquired taxa in faecal microbiota transplantation for recurrent Clostridium difficile infection.](http://paperpile.com/b/e0VSDx/XeD95) [*BMC Med.* **14**, 155 (2016).](http://paperpile.com/b/e0VSDx/XeD95)
6. [Broecker, F. *et al.* Long-term changes of bacterial and viral compositions in the intestine of](http://paperpile.com/b/e0VSDx/SdAIb) [a recovered Clostridium difficile patient after fecal microbiota transplantation. *Cold Spring*](http://paperpile.com/b/e0VSDx/SdAIb) [*Harb. Mol. Case Stud.* **2**, a000448 (2016).](http://paperpile.com/b/e0VSDx/SdAIb)
7. [DuPont, H. L. *et al.* Fecal microbiota transplantation in Parkinson’s disease-A randomized](http://paperpile.com/b/e0VSDx/Yf202) [repeat-dose, placebo-controlled clinical pilot study. *Front. Neurol.* **14**, 1104759 (2023).](http://paperpile.com/b/e0VSDx/Yf202)
8. [Kootte, R. S. *et al.* Improvement of insulin sensitivity after lean donor feces in metabolic](http://paperpile.com/b/e0VSDx/cMmLo) [syndrome is driven by baseline intestinal Microbiota composition. *Cell Metab.* **26**,](http://paperpile.com/b/e0VSDx/cMmLo)

[611–619.e6 (2017).](http://paperpile.com/b/e0VSDx/cMmLo)

1. [van Lier, Y. F. *et al.* Donor fecal microbiota transplantation ameliorates intestinal](http://paperpile.com/b/e0VSDx/hxozz)

[graft-versus-host disease in allogeneic hematopoietic cell transplant recipients. *Sci. Transl.*](http://paperpile.com/b/e0VSDx/hxozz) [*Med.* **12**, eaaz8926 (2020).](http://paperpile.com/b/e0VSDx/hxozz)

1. [Bloom, P. P. *et al.* Fecal microbiota transplant improves cognition in hepatic encephalopathy](http://paperpile.com/b/e0VSDx/LYWAK)

[and its effect varies by donor and recipient. *Hepatology Communications* **6**, 2079–2089](http://paperpile.com/b/e0VSDx/LYWAK) [(2022).](http://paperpile.com/b/e0VSDx/LYWAK)

1. [Damman, C. J. *et al.* Low Level Engraftment and Improvement following a Single](http://paperpile.com/b/e0VSDx/EEW3g) [Colonoscopic Administration of Fecal Microbiota to Patients with Ulcerative Colitis. *PLoS*](http://paperpile.com/b/e0VSDx/EEW3g) [*One* **10**, e0133925 (2015).](http://paperpile.com/b/e0VSDx/EEW3g)
2. [Wilson, B. C. *et al.* Strain engraftment competition and functional augmentation in a](http://paperpile.com/b/e0VSDx/XMh7k) [multi-donor fecal microbiota transplantation trial for obesity. *Microbiome* **9**, 107 (2021).](http://paperpile.com/b/e0VSDx/XMh7k)
3. [Su, L. *et al.* Health improvements of type 2 diabetic patients through diet and diet plus fecal](http://paperpile.com/b/e0VSDx/H11jJ) [microbiota transplantation. *Sci. Rep.* **12**, 1152 (2022).](http://paperpile.com/b/e0VSDx/H11jJ)
4. [Doll, J. P. K. *et al.* Fecal Microbiota Transplantation (FMT) as an Adjunctive Therapy for](http://paperpile.com/b/e0VSDx/wpy9j) [Depression—Case Report. *Front. Psychiatry* **13**, (2022).](http://paperpile.com/b/e0VSDx/wpy9j)
5. [Ma, J. *et al.* Gut microbiota remodeling improves natural aging-related disorders through](http://paperpile.com/b/e0VSDx/IkVUJ) [Akkermansia muciniphila and its derived acetic acid. *Pharmacol. Res.* **189**, 106687 (2023).](http://paperpile.com/b/e0VSDx/IkVUJ)
6. [Halsey, T. M. *et al.* Microbiome alteration via fecal microbiota transplantation is effective for](http://paperpile.com/b/e0VSDx/uE9o6) [refractory immune checkpoint inhibitor-induced colitis. *Sci. Transl. Med.* **15**, eabq4006](http://paperpile.com/b/e0VSDx/uE9o6) [(2023).](http://paperpile.com/b/e0VSDx/uE9o6)
7. [Davar, D. *et al.* Fecal microbiota transplant overcomes resistance to anti–PD-1 therapy in](http://paperpile.com/b/e0VSDx/rpNN) [melanoma patients. *Science* **371**, 595–602 (2021).](http://paperpile.com/b/e0VSDx/rpNN)
8. [Wang, Y. *et al.* Insights into bacterial diversity in compost: Core microbiome and prevalence](http://paperpile.com/b/e0VSDx/LnR1T) [of potential pathogenic bacteria. *Sci. Total Environ.* **718**, 137304 (2020).](http://paperpile.com/b/e0VSDx/LnR1T)
9. [Riquelme, E. *et al.* Tumor Microbiome Diversity and Composition Influence Pancreatic](http://paperpile.com/b/e0VSDx/Edaks) [Cancer Outcomes. *Cell* **178**, 795–806.e12 (2019).](http://paperpile.com/b/e0VSDx/Edaks)
10. [Baruch, E. N. *et al.* Fecal microbiota transplant promotes response in](http://paperpile.com/b/e0VSDx/UpZO) [immunotherapy-refractory melanoma patients. *Science* **371**, 602–609 (2021).](http://paperpile.com/b/e0VSDx/UpZO)
11. [El-Salhy, M. *et al.* Long-term effects of fecal microbiota transplantation (FMT) in patients](http://paperpile.com/b/e0VSDx/D9yFk) [with irritable bowel syndrome. *Neurogastroenterology & Motility* **34**, e14200 (2022).](http://paperpile.com/b/e0VSDx/D9yFk)
12. [Freitag, T. L. *et al.* Minor Effect of Antibiotic Pre-treatment on the Engraftment of Donor](http://paperpile.com/b/e0VSDx/FSTRt) [Microbiota in Fecal Transplantation in Mice. *Front. Microbiol.* **10**, (2019).](http://paperpile.com/b/e0VSDx/FSTRt)
13. [El-Salhy, M., Patcharatrakul, T. & Gonlachanvit, S. Fecal microbiota transplantation for](http://paperpile.com/b/e0VSDx/3LSHQ) [irritable bowel syndrome: An intervention for the 21st century. *World J. Gastroenterol.* **27**,](http://paperpile.com/b/e0VSDx/3LSHQ) [2921–2943 (2021).](http://paperpile.com/b/e0VSDx/3LSHQ)
14. [Halko, N., Martinsson, P.-G., Shkolnisky, Y. & Tygert, M. An algorithm for the principal](http://paperpile.com/b/e0VSDx/LLA0p) [component analysis of large data sets. Preprint at https://doi.org/](http://paperpile.com/b/e0VSDx/LLA0p)[10.48550/arXiv.1007.5510](http://dx.doi.org/10.48550/arXiv.1007.5510) [(2011).](http://paperpile.com/b/e0VSDx/LLA0p)
15. [Wang, X.-W. *et al.* Ecological dynamics imposes fundamental challenges in](http://paperpile.com/b/e0VSDx/zgPLD) [community-based microbial source tracking. *iMeta* **2**, e75 (2023).](http://paperpile.com/b/e0VSDx/zgPLD)
16. [Aggarwala, V. *et al.* Precise quantification of bacterial strains after fecal microbiota](http://paperpile.com/b/e0VSDx/mHqlq) [transplantation delineates long-term engraftment and explains outcomes. *Nature*](http://paperpile.com/b/e0VSDx/mHqlq) [*Microbiology* **6**, 1309–1318 (2021).](http://paperpile.com/b/e0VSDx/mHqlq)
17. [Conover, W. J. Practical Nonparametric Statistics, 3rd Edition.](http://paperpile.com/b/e0VSDx/rgyby)

[https://www.wiley.com/en-us/Practical+Nonparametric+Statistics%2C+3rd+Edition-p-97804](https://www.wiley.com/en-us/Practical%2BNonparametric%2BStatistics%2C%2B3rd%2BEdition-p-9780471160687) [71160687](https://www.wiley.com/en-us/Practical%2BNonparametric%2BStatistics%2C%2B3rd%2BEdition-p-9780471160687)[.](http://paperpile.com/b/e0VSDx/rgyby)
